# An integrative analysis of post-translational histone modifications in the marine diatom *Phaeodactylum tricornutum*

**DOI:** 10.1186/s13059-015-0671-8

**Published:** 2015-05-20

**Authors:** Alaguraj Veluchamy, Achal Rastogi, Xin Lin, Bérangère Lombard, Omer Murik, Yann Thomas, Florent Dingli, Maximo Rivarola, Sandra Ott, Xinyue Liu, Yezhou Sun, Pablo D. Rabinowicz, James McCarthy, Andrew E. Allen, Damarys Loew, Chris Bowler, Leïla Tirichine

**Affiliations:** Ecology and Evolutionary Biology Section, Institut de Biologie de l’École Normale Supérieure (IBENS), CNRS UMR8197 INSERM U1024, 46 rue d’Ulm, 75005 Paris, France; Institut Curie, PSL Research University, Centre de Recherche, Laboratoire de Spectrométrie de Masse Protéomique, 26 rue d’Ulm, 75248 Cedex 05 Paris, France; Institute for Genome Sciences (IGS), University of Maryland School of Medicine, Baltimore, MD 21201 USA; J. Craig Venter Institute, 10355 Science Center Drive, San Diego, CA 92121 USA; Scripps Institution of Oceanography, Integrative Oceanography Division, University of California, San Diego, CA 92093 USA; Present address: BESE Division, Center for Desert Agriculture, King Abdullah University of Science and Technology, Thuwal, 23955-6900 Saudi Arabia; Present address: State key lab of Marine Environmental Science, Xiamen University, Xiamen, 361005 China; Present address: Instituto de Biotecnología, CICVyA, Instituto Nacional de Tecnología Agropecuaria (INTA Castelar), CC 25, Castelar, B1712WAA Argentina

## Abstract

**Background:**

Nucleosomes are the building blocks of chromatin where gene regulation takes place. Chromatin landscapes have been profiled for several species, providing insights into the fundamental mechanisms of chromatin-mediated transcriptional regulation of gene expression. However, knowledge is missing for several major and deep-branching eukaryotic groups, such as the Stramenopiles, which include the diatoms. Diatoms are highly diverse and ubiquitous species of phytoplankton that play a key role in global biogeochemical cycles. Dissecting chromatin-mediated regulation of genes in diatoms will help understand the ecological success of these organisms in contemporary oceans.

**Results:**

Here, we use high resolution mass spectrometry to identify a full repertoire of post-translational modifications on histones of the marine diatom *Phaeodactylum tricornutum*, including eight novel modifications. We map five histone marks coupled with expression data and show that *P. tricornutum* displays both unique and broadly conserved chromatin features, reflecting the chimeric nature of its genome. Combinatorial analysis of histone marks and DNA methylation demonstrates the presence of an epigenetic code defining activating or repressive chromatin states. We further profile three specific histone marks under conditions of nitrate depletion and show that the histone code is dynamic and targets specific sets of genes.

**Conclusions:**

This study is the first genome-wide characterization of the histone code from a stramenopile and a marine phytoplankton. The work represents an important initial step for understanding the evolutionary history of chromatin and how epigenetic modifications affect gene expression in response to environmental cues in marine environments.

**Electronic supplementary material:**

The online version of this article (doi:10.1186/s13059-015-0671-8) contains supplementary material, which is available to authorized users.

## Background

Eukaryotic histones are small proteins involved in the formation of nucleosomes, the basic repeating unit of chromatin comprising a histone core around which approximately 146 base pairs of DNA wrap, allowing it to be packaged into the nucleus [1]. The histone core consists of a histone octamer comprising two copies of histone H2A and H2B dimers and one copy of a histone H3-H4 tetramer, all linked to the next nucleosome by the histone linker H1, which appears to be an essential element for stabilizing the folding and condensation of chromatin [2]. Histones are substrates for a diverse range of post-translational modifications (PTMs). These PTMs can occur alone or in a combinatorial fashion (known as the ‘histone code’) and define dynamic transitions between active and silent chromatin states that co-regulate important biological processes [3]. PTMs occur primarily on the N-terminal tails of histones but also on their globular domains and their C-termini, and include methylation, acetylation, phosphorylation, ubiquitination, sumoylation, citrullination, ADP-ribosylation, hydroxylation, and crotonylation of specific residues [4]. While histone acetylation is generally associated with gene activation, methylation of specific lysine residues can be associated with either active or silent chromatin states depending on the residue that is modified, and whether it is mono-, di-, or tri-methylated. Furthermore, histone phosphorylation is involved in transcriptional regulation of a wide range of biological processes, such as mitosis, DNA replication and damage repair, stress responses, and activation of transcription.

Histones are one of the most highly conserved groups of proteins throughout evolution, highlighting their important role in living organisms. They have been found in almost all eukaryotes so far examined, and although they are not found in bacteria, they do occur in some Archaea [5], indicating their ancient origin. They have been extensively studied in several model organisms, including human, *Drosophila*, yeast and *Arabidopsis*. However, little is known about their role in genome organization in phylogenetically distant groups of eukaryotes beyond the Opisthokonta (including metazoans and fungi) and the Archaeplastida (higher plants, green and red algae).

The chromalveolate group is one of the most diverse groups of eukaryotes, and includes ciliates and dinoflagellates (members of the Alveolata), as well as oomycetes and diatoms (representatives of the Stramenopila, also known as Heterokonta) [6]. Very little is known about the genome structure of these organisms. Ciliates, for example, show a peculiar genome organization reminiscent of the germline-soma distinction in other eukaryotes, with a macronucleus, where transcription of protein coding genes takes place, and a germline micronucleus, which remains silenced [7]. This diversity in genome organization is also seen in dinoflagellates, whose chromosomes are attached to the nuclear membrane and lack canonical histones [8].

Diatoms (Bacillariophyta) are one of the major groups of chromalveolates. Although chronically understudied from a molecular perspective, they are a fundamental component of phytoplankton in most aquatic ecosystems, and are believed to contribute around 40 % of primary production in marine ecosystems [9]. Whole genome sequencing of two marine diatoms, *Thalassiosira pseudonana* and *Phaeodactylum tricornutum*, has revealed their unusual genomic composition, proposed to be a result of endosymbiotic gene transfers involving green and red algae, as well as a significant amount of horizontal gene transfer from bacteria [10]. The combination of genes from different origins has attributed them with novel metabolic capacities for photosynthetic organisms, such as fatty acid oxidation pathways and a urea cycle centered in their mitochondria [11]. These pathways are central hubs of diatom primary metabolism and are also used for diatom-specific processes, such as the construction of their silicified cell walls, known as frustules [11, 12].

Diatoms are remarkably successful organisms with a broad distribution in contemporary oceans and with a well-known capacity to adapt rapidly and outcompete other phytoplankton when favorable conditions arise [13], suggesting that epigenetic regulation mechanisms might contribute to their success. We therefore used high accuracy mass spectrometry (MS) to draw a comprehensive landscape of PTMs in *P. tricornutum*. Using chromatin immunoprecipitation (ChIP), we generated whole genome maps of five PTMs and compared their distributions with a previously generated DNA methylation landscape [14]. Finally, we demonstrate the dynamic nature of the chromatin code by revealing changes in response to nutrient limitation.

## Results

### Identification of histone PTMs using mass spectrometry

The *P. tricornutum* genome encodes 14 histone genes dispersed on 5 of the 34 chromosome scaffolds characterized previously [15]. Most are found in clusters of two to six genes, as seen in other, albeit not all, eukaryotes such as the ciliates *Stylonychia lemnae*, *Tetrahymena thermophila* and related species [16, 17]. The phylogenetic clustering of *P. tricornutum* histones in doublets of H3-H4 and H2A-H2B reflects their similar evolutionary history, which involves the progressive diversification and differentiation of the four core histone families through a mechanism of recurrent gene duplication [18] (Additional file 1). While histones H4 and H2B are highly conserved, the *P. tricornutum* genome encodes one variant of histone H1, and two variants of each histone H3 and H2A. Further *in silico* analysis revealed that the 27 Mb genome contains a plethora of histone-modifying enzymes, including histone acetyl transferases and deacetylases, and methyl transferases and demethylases [19].

**Fig. 1:**
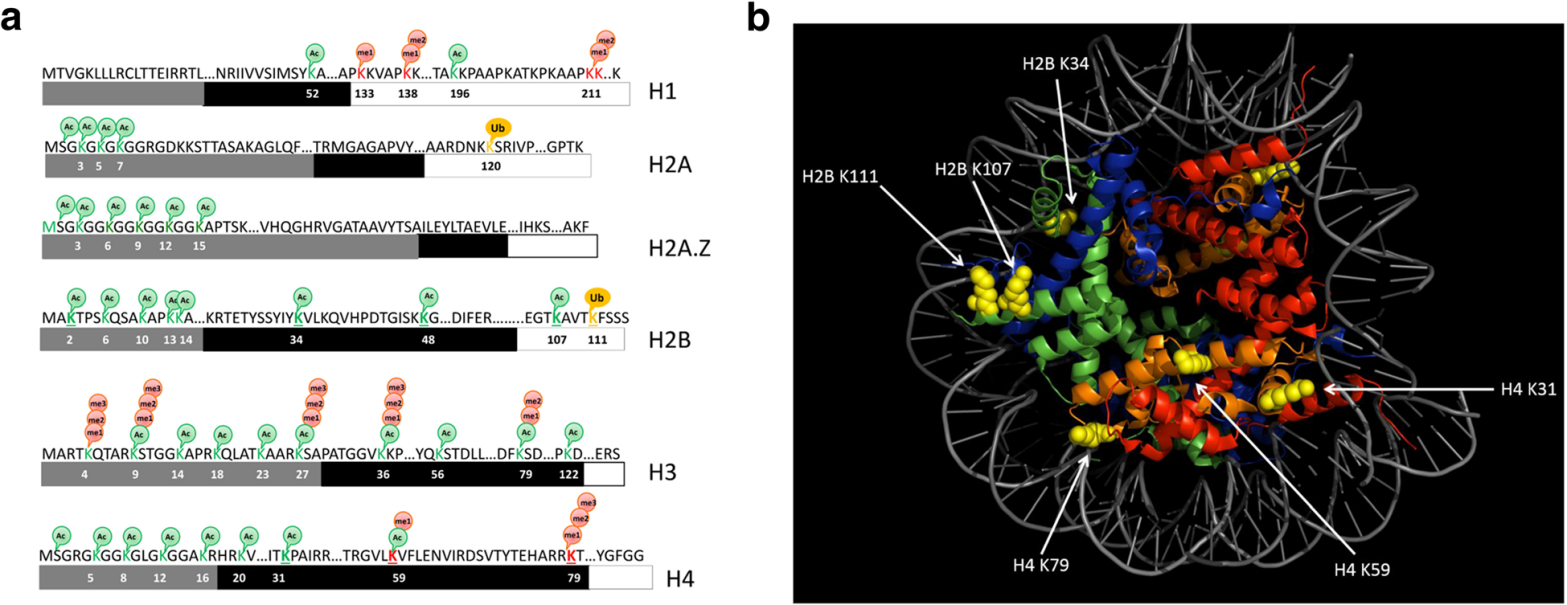
Histone PTMs in *P. tricornutum*. **a** Sites of PTMs of linker, core and variant histones identified in this work. Amino acid residue number is indicated below the peptide sequence. Gray, black and white boxes indicate N-terminal, globular core and C-terminal domains, respectively. Because K211 and K212 are contiguous, their methylation state cannot be discriminated. Me2 can be located on K211 or K212 of histone H1. H3K9me2 and H3K9me3 were detected by western blot. Novel modifications are underlined. **b** Novel histone modifications are modeled on the crystal structure of the nucleosome (Protein Data Bank file 3A6N). The histone proteins are shown in ribbon diagram with histone H2A in red, H2B in orange, H3 in blue, and H4 in green. The DNA helix is shown in gray. Modified residues are visible as yellow spheres. The image was generated using the program Pymol [24]

To identify histone PTMs in *P. tricornutum*, we used high-accuracy MS combined with different enzyme digests with purified histone preparations [20]. Subsequent manual inspection and validation of MS data resulted in a high level of confidence in discriminating between modified sites with the same nominal masses (see Materials and methods for details). In total we identified 62 PTMs on the core and variant histones, among which eight are novel or have been identified previously in only one species (Fig. 1a). As expected, most PTMs are on the protruding N-terminal tails, although a substantial number of modified sites were also detected on the globular domains (Fig. 1a; Additional file 2). A range of lysine residues exhibited multiple modifications comprising mono-, di- and tri-methylation, acetylation and mono-ubiquitination, many of which are shared with mammals and plants. However, the positions of some modified sites were not conserved, such as the ubiquitination of lysine 111 of histone H2B. N-terminal acetylation was observed on H2A.Z and H4, where the initial methionine was lost during protein processing and the subsequent serine residue was acetylated. We could not detect either di- or tri-methylation of lysine 9 of histone H3 despite the presence of the histone modifying enzyme of the SuVar family. However, we have shown the presence of both modifications by western blot in a previous work [21]. Of note, neither H3K9me2 nor H3K9me3 were detectable by MS in *Arabidopsis* despite their occurrence in vivo [22, 23]. Although several arginine methylases are encoded in the *P. tricornutum genome* [19], methylation of arginine was not detected, which might be due to its low abundance. Some modifications were shared only with metazoans and not plants, such as mono-, di- and tri-methylation of lysine 79 of histone H4. Besides these novel PTMs, we identified five additional unique modifications, acetylation of lysines 31 and 59 of histone H4 as well as acetylation of lysines 2, 34 and 107 of H2B (Fig. 1a, b).

### Genome-wide distribution of H3K4me2, H3K9me2, H3K9me3, H3AcK9/K14 and H3K27me3

**Fig. 2:**
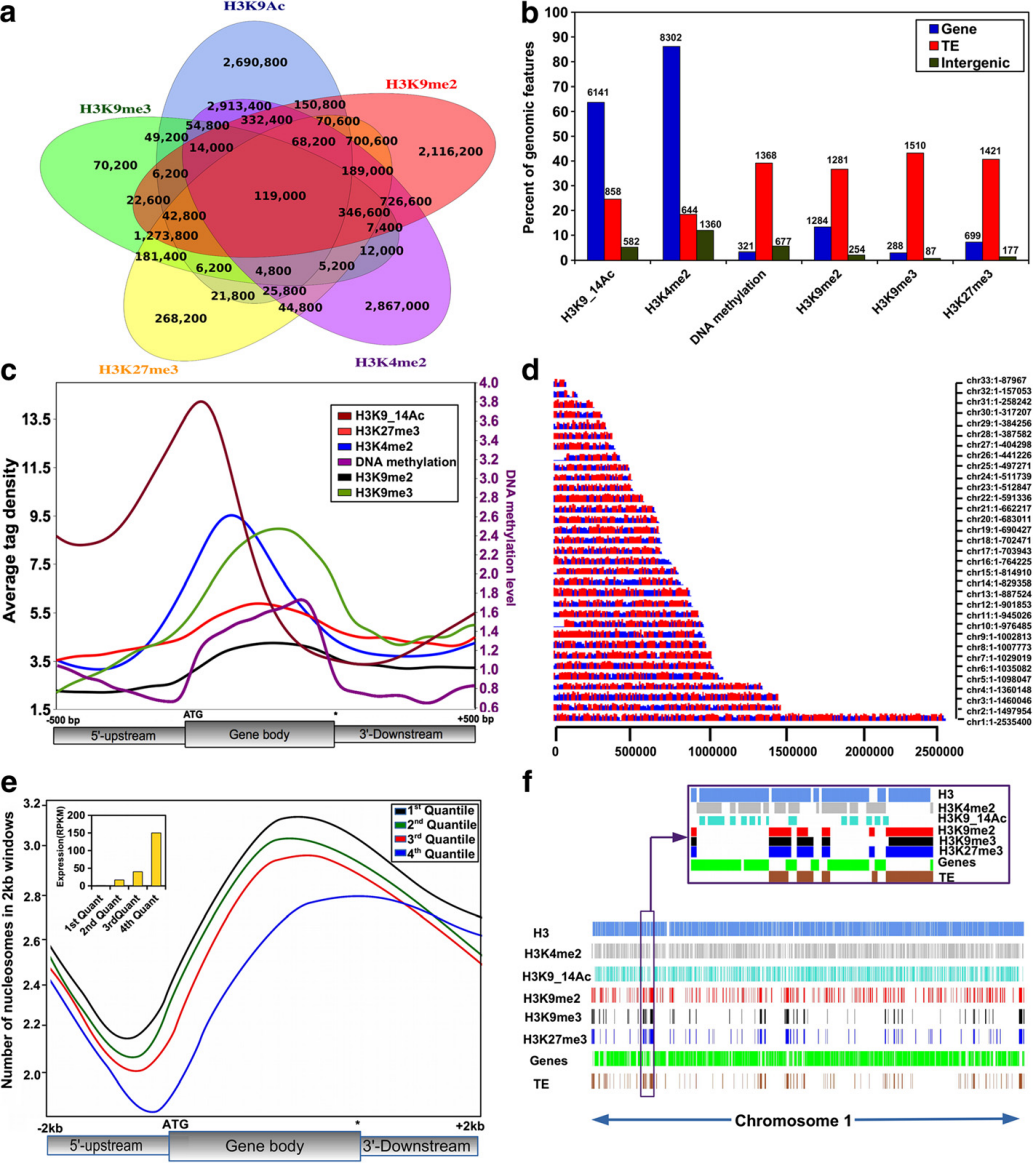
Distribution of ChIP-Seq signal peaks for H3K4me2, H3K9_14Ac, H3K9me2, H3K9me3, and H3K27me3. **a** Venn diagram showing the overlap (in base pairs) of histone marks with each other. **b** Percentage of genomic features (genes, TEs, and intergenic regions) found enriched for each of the marks. The numbers above each bar refer to total number of peaks overlapping genes, TEs or intergenic regions. **c** Enrichment profile of H3K4me2, H3K9_14Ac, H3K9me2, H3K9me3, H3K27me3 along genes (upstream 500 bp, coding region, downstream 500 bp). Average tag density is the number of sequence reads per gene. Note that the small rise in enrichment seen in the flanking regions (both at 5′ and 3′ ends) is a result of the presence of nearby genes in the densely packed genome. **d** Genome-wide nucleosome distribution. The color code refers to the level of enrichment, blue being low and red high. **e** Nucleosome occupancy along genes and flanking regions and its correlation with gene expression quantiles (Reads Per Kilobase Per Million, *RPKM*). **f** A snapshot of chromosome 1, showing the six epigenetic modifications within genes and TEs

The identification of several histone PTMs by MS provides an opportunity to investigate their biological role and significance in *P. tricornutum*. We chose to focus our analyses on a few histone marks that have known functions in transcriptional activation or repression so as to draw inferences of possible regulatory roles in diverse biological processes. Genome-wide mapping of five histone marks (H3K4me2, H3K9me2, H3K9me3, H3K27me3 and H3AcK9/14) as well as nucleosome occupancy was generated by ChIP followed by deep sequencing (ChIP-Seq; see Materials and methods). Two biological replicates were sequenced for each mark and statistical tests showed a positive correlation between replicates (multiple hypothesis testing with 10 % false discovery rate, FDR *p*-value <0.005), thus validating the accuracy of the data. We generated high coverage maps with 4 to 38 million reads that uniquely mapped to the *P. tricornutum* genome (Additional file 3). Almost 40 % of the genome was marked by the five histone modifications. H3K4me2-, H3K9/14Ac-, H3K9me2-, H3K9me3- and H3K27me3-marked regions covered ~29 %, ~25 %, ~25 %, ~8 % and ~14 % of the genome, respectively (Additional file 4). The genome shows regions marked by at least one histone modification, and a total of 119,000 genomic regions — genes, intergenic regions, transposable elements (TEs) (1,228,620 bp) — are shared between all the marks (Fig. 2a).

We further investigated the distribution of histone modification peaks on genes, TEs and intergenic regions. Based on the number of modified domains, we found more enriched domains within genic regions than on TEs for all the marks except H3K9me3 (Additional file 5). Furthermore, a significant percentage of H3K4me2- and H3K9/14Ac-modified domains lay within intergenic regions.

A systematic analysis of the locations of H3K4me2- and H3K9/14Ac-marked regions revealed a highly significant correlation between their location and the presence of annotated genes. Around 86 % and 63 % of annotated genes were found to be associated with H3K4me2 and H3K9/14Ac, respectively, while only 605 and 741 TEs, respectively, were marked (Fig. 2b). In stark contrast with these two marks, H3K9me2 and H3K9me3 were found associated principally with annotated TEs. A total of 1281 and 1510 TEs were found to be marked with H3K9me2 and H3K9me3, respectively, even though a significant number of genes were also marked by H3K9me2 (Fig. 2b). Compared with H3K9me2, H3K27me3 was even more highly enriched on TEs, which is surprising and unusual compared with other organisms for which this mark has been profiled [25, 26]. A total of 1421 out of 3493 TEs were associated with H3K27me3 while only 700 genes were marked with it (Fig. 2b).

### Distribution patterns of H3K4me2, H3K9me2, H3K9me3, H3AcK9/K14 and H3K27me3 on genes

To investigate the enrichment patterns of each histone modification on genes, we examined the average enrichment of the five histone marks on predicted genes over their entire coding sequence (CDS), and within regions 500 bp upstream and downstream of the CDS. The enrichment of H3K9/14Ac and H3K4me2 peaks significantly close to the 5′ end of CDSs, with a sharper peak for H3K9/14Ac (Fig. 2c).

To assess whether the genes marked with specific histone modifications are enriched in specific functional categories, we performed a gene ontology (GO) classification. The genes marked by H3K4me2 (6047; 62.8 % of annotated genes) are enriched in the structural constituent of ribosome GO category compared with the rest of the genes in the genome while those marked by H3K27me3 (700; 7 % of annotated genes) are enriched in protein kinase activity, cAMP-dependent protein kinase, phosphotransferase, and diamine N-acetyl transferase GO categories. H3K9me2- and H3K9me3-marked genes (218; 2.3 % of annotated genes) were found to be enriched in hydrolase, ATPase, inorganic cation transmembrane transport, nucleoside tri-phosphatase activity, helicases, and structural constituent of cytoskeleton GO categories. H3K9/14ac marked genes were not enriched in any particular GO category (Additional file 6).

### Genome-wide mapping of H3K4me2, H3K9me2, H3K9me3, H3AcK9/K14 and H3K27me3 on TEs

*P. tricornutum* TEs contain class I elements, including long terminal repeat retrotransposons (LTR-RTs; Copia), relics of non LTR-RT retrotransposon-like elements, and a few copies of class II transposons, including Piggybac, Tpase-like, and MuDR-like elements [27]. Among them, 1350 (38.7 %), 1510 (43 %) and 1421 TEs (41 %) were marked by H3K9me2, H3K9me3 and H3K27me3, respectively (Figure S5A in Additional file 7). Most of these marked TEs belong to Copia-type elements. A total of 1158, 1163 and 1281 Copia TEs were found to be marked by H3K9me2, H3K9me3 and H3K27me3, respectively, and most of them are not transcribed (Figure S5A, B in Additional file 7). As for H3K9/14Ac and H3K4me2, which have a transcription activating effect on TEs (Figure S5B in Additional file 7), only 858 and 644 TEs were marked, respectively, and most of these were found to be simple repeats (n = 657 and n = 337, respectively). Overall, a significant fraction of potentially active Copia TEs were found associated with H3K9me2, H3K9me3 and H3K27me3, which implies that these marks may regulate the transcriptional activation of TEs, especially Copia-type TEs, which appear likely to have amplified recently in the genome of *P. tricornutum* [27].

### Nucleosome occupancy in the *P. tricornutum* genome

Nucleosome occupancy plays an important role in cellular processes, allowing selective access to the DNA by regulatory elements such as transcription factors [28]. To assess the relative size of nucleosomes, we performed micrococcal nuclease (MNase) digestion of isolated nuclei using increasing concentrations of MNase. Separation of the digested product in agarose gels showed a major band around 150 bp, which is a similar size to that found in plant and metazoan nucleosomes (Figure S6A in Additional file 8).

To evaluate the relative nucleosome occupancy over the *P. tricornutum* genome, we performed ChIP-Seq with an antibody against the unmodified carboxyl terminus of histone H3. Close to 60 % of the genome was found to be occupied by nucleosomes, with densely packed segments interspersed by nucleosome-depleted regions (Fig. 2d). Most of the nucleosomes fall within exons and a significant number cover TEs and intergenic regions (Figure S6B in Additional file 8). We further examined nucleosome distribution over CDSs, upstream of the transcription start site and downstream of the stop codon, and assessed how this correlated with the expression state of the genes. Our data show that nucleosome depletion occurs around 150 bp upstream of the transcription start site for genes that have high expression quantiles while nucleosome density increases over gene bodies and drops towards the 3′ end for all genes, regardless of their expression quantile, which is consistent with what has been reported in other species (Fig. 2e).

Previous work in other organisms identified nucleosome positions based on DNA sequence motifs [29]. We therefore tested whether DNA sequence-guided nucleosome positioning is of relevance in *P. tricornutum*. We found that GC and CG are dinucleotide sequences where nucleosomes are preferentially positioned whereas AA, TA and TT can be considered nucleosome-excluding sequences and rather tend to peak outside the nucleosomes (Figure S6C in Additional file 8), as observed in other species [29].

### Correlation of chromatin marks with gene expression

A representative genomic region of chromosome 1 is shown in Fig. 2f to demonstrate the general distribution of the five histone marks along with histone H3 on genes and TEs. It shows that, in general, genes are co-marked by H3K4me2 and acetylation while TEs tend to be marked by H3K9me2, H3K9me3 as well as H3K27me3.

**Fig. 3:**
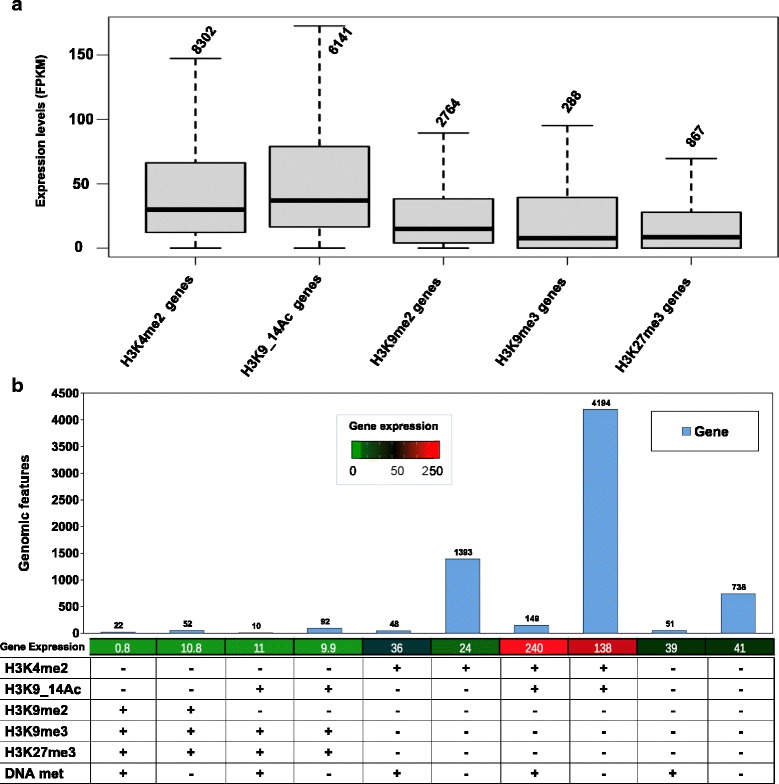
Comparison of the presence of histone marks with gene expression. **a** Boxplots showing the gene expression profiles correlating with each histone mark. Gene expression was quantified in standard growth conditions using RNA-Seq data. Around 85 % of genes are expressed and quantified as fragments per kilobase of exons per million reads mapped (*FPKM*). Number of genes is indicated above each bar. **b** Combinatorial effects of histone modifications and DNA methylation on gene expression. FPKM values are indicated below each bar

To explore the relationship between gene expression and each of the five histone marks, the mRNA levels of genes were assessed genome-wide using RNA-Seq in the same growth conditions that were used for the cells used for chromatin analyses. The genes marked by both H3K4me2 and H3K9/14Ac showed the highest average expression levels, and the latter showed the largest variation in expression (Fig. 3a). By contrast, both H3K9me3- and H3K27me3-marked genes displayed the lowest gene expression levels, indicating their association with repressed genes in *P. tricornutum*, consistent with previous studies in different organisms [30–32]. H3K9me2 also displayed a moderate repressive effect on genes. Taken together, these observations suggest that H3K4me2 and H3K9/14Ac represent general marks for expressed genes, whereas H3K9me3, H3K27me3 and H3K9me2 appear to associate with repressed genes.

Combination of two or more histone modifications is known to have an impact on gene regulation beyond those of individual marks [33, 34]. We therefore examined whether particular combinations of histone PTMs may influence transcriptional regulation of genes in *P. tricornutum*. A large number of genes marked with both H3K4me2 and H3K9/14Ac were significantly correlated with high levels of expression, further supporting the role of these two marks in the activation of gene expression, while co-occurrence of H3K27me3 with either H3K9me2 or H3K9me3 correlated with a low level of gene expression, indicating the repressed state of these co-marked genes (Fig. 3b).

We further correlated DNA methylation from previously published work [14] with histone PTMs and examined gene expression patterns of marked genes. As noted above, genes co-marked with H3K4me2 and H3K9/14Ac were upregulated, and this was largely unaffected by the presence or absence of DNA methylation (Fig. 3b). DNA methylation had no major effect on expression patterns, except on 48 genes labeled with H3K4me2, which were significantly highly expressed compared with other H3K4me2 genes that were not methylated (Fig. 3b). We have already shown that DNA methylation has no significant effect on expression of genes except when they are extensively methylated [14]. Furthermore, the genes co-marked by H3K4me2 and DNA methylation might have additional activating histone marks that we did not investigate in this study, such as H3K4me3, H3K36me3 and H3AcK27, which could explain the significant increase in gene expression.

Combination of the five histone marks with DNA methylation defined three main chromatin states (CSs): CS1, which is activating and correlates with the presence on genes of H3 acetylation and H3K4me2; CS2, which is repressive and is defined predominantly by H3K27me3, H3K9me3 and H3K9me2; and CS3, which combines activating and repressive marks with an intermediate expression level of genes. It should also be noted that a significant number of genes are not marked and may contain histone marks that were not investigated in this study (Fig. 3b).

**Fig. 4:**
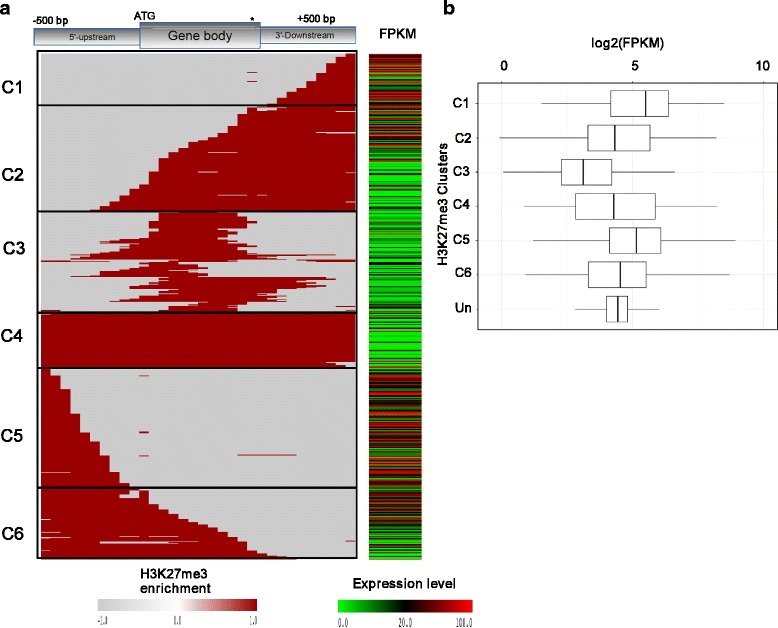
Clustering analysis of H3K27me3 over genes and correlation with gene expression. **a** H3K27me3 enrichment is shown in red while absence is represented in gray. Gene expression levels are shown as a heat map with red reflecting high (expression level FPKM [fragments per kilobase of exons per million reads mapped] greater than 20) and green representing low expression (expression level FPKM between 0 and 20). **b** Expression boxplots for each H3K27me3 enrichment cluster. *Un* refers to genes unmarked by H3K27me3

H3K27me3 is characterized by a broad distribution pattern over several kilobases in animals, while plants such as *Arabidopsis* display shorter H3K27me3-marked domains restricted mainly to transcribed regions [32, 36]. Although a photosynthetic organism, *P. tricornutum* shows an animal-like distribution pattern of H3K27me3, perhaps suggesting similar mechanisms of deposition and transcriptional regulation of genes. Polycomb repressive complex 2, containing four proteins, methylates H3K27me via the SET domain of its subunit enhancer of zeste [37]. The PRC2 complex is widely distributed among plants, metazoans and algae but appears to be absent from the yeast species *Saccharomyces cerevisiae* and *Schizosaccharomyces pombe* [38]. Considering the presence of different clusters within the wide H3K27me3 domains reported in different species [26, 31, 37] and the lack of knowledge about the distribution pattern of this mark in single-celled organisms, we assessed in more detail the pattern of H3K27me3 enrichment over genes. Interestingly, we could distinguish six different profiles. The first cluster of genes shows a distinct enrichment over the region 500 bp downstream of the stop codon (C1), while the other clusters target the gene body and 500 bp downstream (C2), the gene body only (C3), the entire gene length (C4), only the region 500 bp upstream of the TSS (C5), and 500 bp upstream and the gene body (C6) (Fig. 4a). When correlated with expression data, only four clusters (C2, C3, C4 and C5) correlate clearly with repressed genes while clusters C1 and C6 show positive correlations with gene expression compared with unmarked genes, suggesting a different or a diversified role of H3K27me3 in *P. tricornutum* which is known to be repressive (Fig. 4b). We performed a homology estimation analysis (see Materials and methods) and found out that out of 700 H3K27me3-marked genes, 39 % have no homologs with known function and nearly 20 % are found only in *P. tricornutum* (Additional file 9). Of note, none of the other investigated marks showed such a clustering pattern.

### Changes in chromatin marks in response to nutrient-limiting conditions

To gain insights into the dynamic nature of the *P. tricornutum* epigenome in response to an environmental cue, we analyzed the impact of nitrate depletion. Nitrate is an important nutrient in marine ecosystems and its appearance in surface waters, e.g., following upwelling events, is often associated with diatom proliferation [13]. We specifically examined three histone modifications (H3K4me2, H3K9/14Ac and H3K9me3) using Chip-seq, as well as DNA methylation by bisulfite deep sequencing. We also assessed gene expression changes by RNA-Seq.

**Fig. 5:**
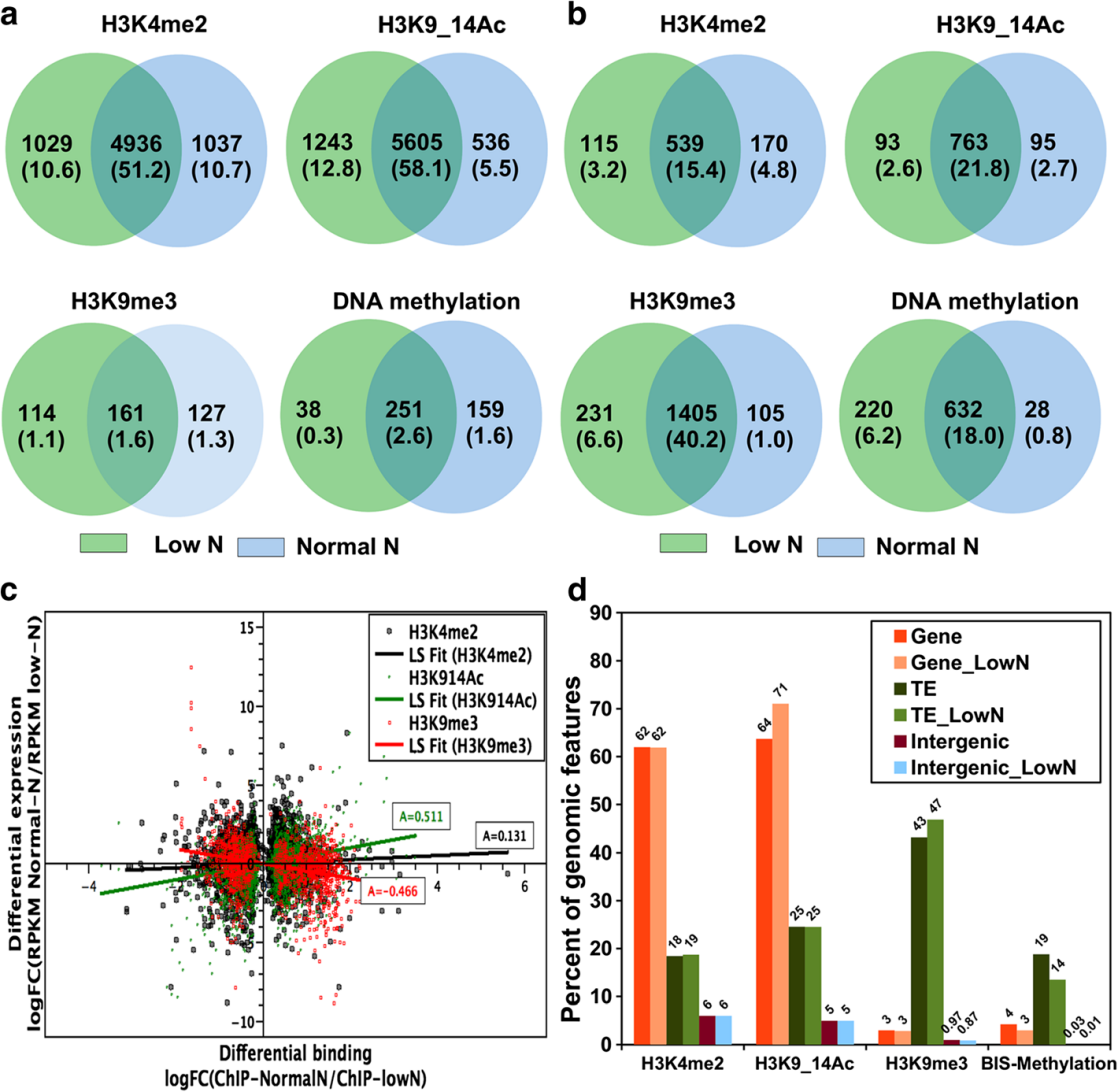
Dynamic changes in chromatin marks in nitrate replete and limiting conditions. **a** Venn diagrams showing overlap of different histone marks on genes in nitrate replete (*Normal N*) and nitrate-limiting (*Low N*) conditions. **b** Venn diagrams showing overlap of different histone marks on TEs in nitrate replete and nitrate-limiting conditions. Percentages of differentially marked genes and TEs are indicated between parentheses. **c** Comparison of levels of ChIP binding and expression levels. Fold change of ChIP binding (differential binding) is calculated from the number of reads in the peak region using diffReps. Fold change in expression level is calculated from the levels of RPKM using the Cuffdiff [78]. Pearson correlation values between differentially marked and expressed genes under the two conditions (normal and low nitrate growth conditions) are marked along the trend line. **d** Number of genome features (genes and transposable elements) marked by each of the three histone marks, as well as DNA methylation. Percentage was derived using the known genome feature annotations. Number of genes, TEs and intergenic regions under normal or low nitrate are indicated above each bar

In parallel with the reduced growth rate and chlorotic phenotype observed during nitrate limitation (Additional file 10), the number of genes that lost or gained histone marks and/or DNA methylation was noteworthy, in particular H3K9/14 acetylation and H3K4me2 (Fig. 5a). These changes were more prominent on genes than on TEs, except for H3K9me3 and DNA methylation, which showed an opposite profile, indicating that TEs are probably tightly regulated by these two marks, which show repressive effects in response to stress (Fig. 5b). Almost 20 % of H3K4me2-marked genes lost this mark under nitrate depletion while ~16 % of H3K9/14Ac-free genes gained this mark. The loss of both H3K9me3 and DNA methylation was even more significant (31 % and 35 %, respectively). As expected, the chromatin profiles of most genes and TEs remained the same, suggesting that only certain sets of genes and TEs were affected by nitrate limitation. Very few intergenic regions were differentially marked between both conditions, suggesting they have a minor role in gene regulation in response to nitrate limitation (data not shown).

**Fig. 6:**
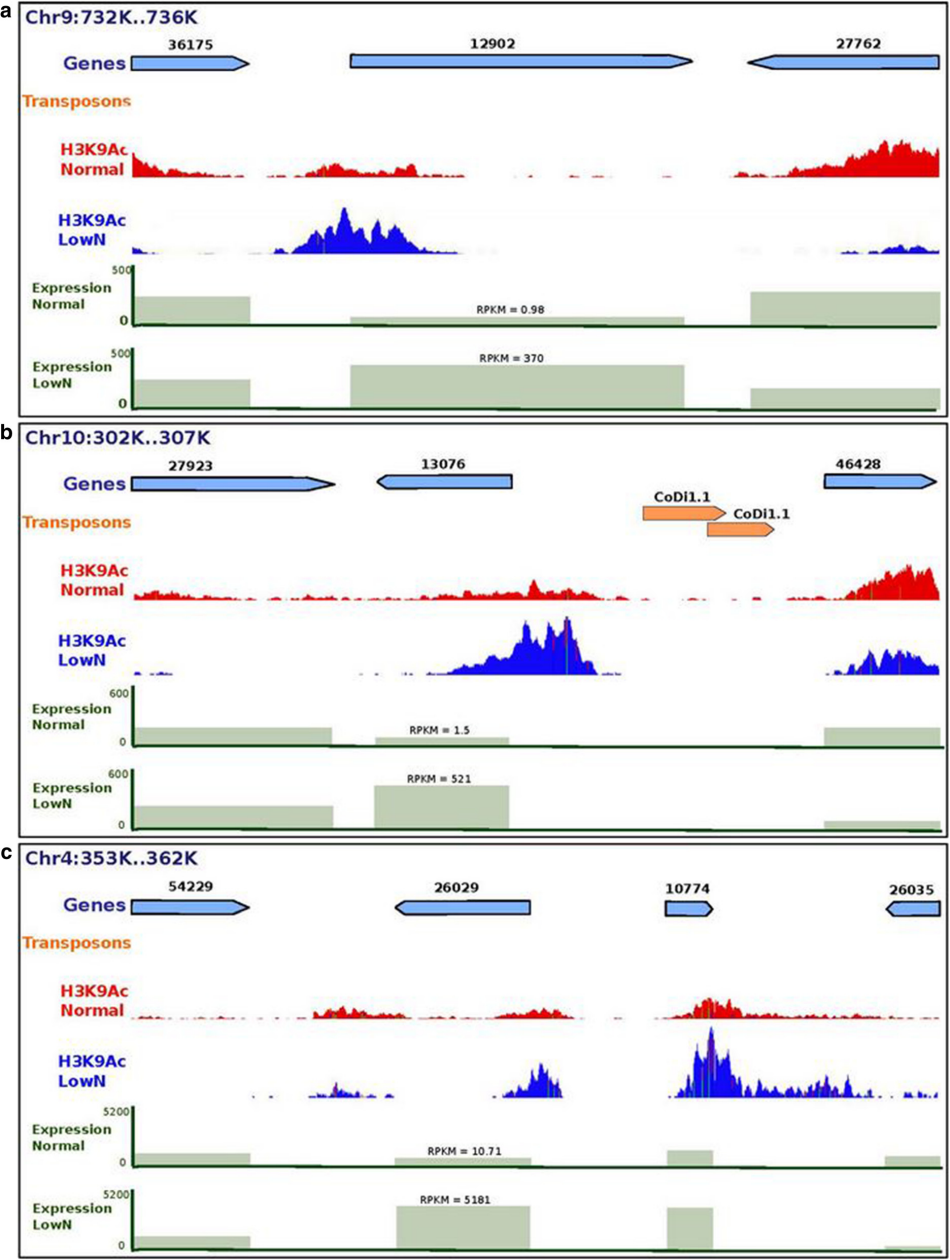
Snapshots of genes differentially marked and regulated under nitrate depletion. Ferredoxin nitrite reductase (12902) (**a**), a nitrite transporter (13076) (**b**) and a nitrate transporter (26029) (**c**) as well as chloroplast ribosomal proteins are shown with higher levels of acetylation and expression under low nitrate conditions

To determine whether these patterns of histone modifications were correlated with changes in transcriptional regulation, we used a global quantification approach to examine the link between differentially marked genes and the fold change in expression under nitrate replete and limiting conditions. The overall effect of differential marking by both acetylation and H3K4me2 had a positive effect on gene expression, while the differential marking of H3K9me3 showed a rather repressive effect on gene expression (Fig. 5c, d). Many genes gained acetylation and/or H3K4me2 under low nitrate and therefore became upregulated, as did those that lost H3K9me3. This analysis pinpointed genes involved in nitrate metabolism, e.g., ferredoxin-dependent nitrite reductase (Pt12902), and nitrite (Pt13076) and nitrate (Pt26029) transporters, which were all acetylated under nitrate starvation, which correlated with their transcriptional upregulation (Fig. 6; Additional file 11).

To gain further insights into the functional categories of genes that were differentially marked and regulated under low nitrate, we performed a GO classification as well as a Mapman analysis and found that, as expected, there is an enrichment in genes encoding proteins involved in nitrate metabolic pathways (nitrate transport, reduction and assimilation) as well as genes involved in lipid transport and metabolism, and stress response in conditions of nitrate limitation (Additional files 11, 12, 13, 14, and 15). Other genes involved in different pathways related to nitrate availability were also found to be marked and expressed differentially. For example, genes encoding phytoene desaturase-like3 (Pt15806), known to be involved in carotenoid biosynthesis, coproporphyrinogen III oxidase (Pt10640), involved in heme and chlorophyll synthesis, as well as several proteins involved in light harvesting (Pt14442, Pt25168, Pt22956, Pt22395, Pt47697) gain H3K4me2 under low nitrate, while genes encoding an ATP binding protein (Pt46431) or encoding secondary metabolite biosynthesis components (Pt16295) gained H3K9me3. Stress response genes were also found to be differentially regulated. Most of the genes that gain or lose DNA methylation encode proteins involved in catalytic activities and metabolism. A few other genes indirectly related to nitrate depletion showed differential regulation in response to nitrate limitation, including Pt15815, which encodes an ortholog of a pyrophosphatase-energized proton pump (involved in auxin transport in plants), whose enhanced activity was shown to improve nitrogen uptake in roman lettuce [39], and Pt51183, which encodes a CREG1 ortholog, involved in cellular repression of transcription. Interestingly, many genes of unknown function lost one of the marks, in particular acetylation under low nitrate conditions. Among these genes, many were found to be diatom specific — 30 % of H3K9me3-, 36 % of H3AcK9/14- and 40 % of H3K4me2-marked genes; these represent 9 %, 9.6 % and 3.6 %, respectively, of the *Phaeodactylum* genome, suggesting particular pathways have been recruited by diatoms to survive nitrate depletion in the oceans.

## Discussion

We report here a comprehensive analysis of histone PTMs in the model diatom *P. tricornutum* using MS and ChIP-Seq. MS analysis revealed a large conservation of histone modifications but also new ones, thus expanding the list of histone PTMs in eukaryotes. Most of the histone modifications showed similarities to those of plants and mammals, including acetylation of several lysines on the N-terminal tails of histones H2A, H2B, H3 and H4 and mono-, di- and tri-methylation of lysines 4, 9, 27 and 36 of histone H3, suggesting their role in transcriptional regulation of many biological processes.

MS analysis revealed eight less-characterized modifications, namely acetylation of lysine 59 of histone H4, which was instead reported to be methylated in bovine calf thymus histones [40], and acetylation of lysine 31 of histone H4 (reported only in *Toxoplasma* [41], although not confirmed in an independent study [42]). In addition, we also detected acetylation of lysines 2, 34 and 107 of H2B, which are reported for the first time in this study, as well as the ubiquitination of lysine 111 of the same histone. Due to their accessibility, histone tail modifications have been widely studied and have been shown to act primarily through altering the ability of non-histone proteins to interact with chromatin. On the other hand, less-studied histone modifications in the core domain, such as the novel ones identified in this work, are likely to exert their function through mechanisms that are distinct from those reported for histone tail modifications. H2BAcK34, H4AcK31, H4AcK59, H4K79me1, H4K79me2 and H4K79me3 are located on the lateral surface of the nucleosome (Fig. 1b), suggesting a primary function in the regulation of histone-DNA interactions. To explain how these modifications might alter chromatin structure, a model has been proposed whereby a chromatin remodeling activity acts on the nucleosomes to alter histone-DNA interactions, thereby exposing sites on the lateral surface which in turn become modified and alter the mobility of nucleosomes. This altered mobility can either lead to changes in the accessibility of specific DNA sequences or changes in higher order chromatin structure [39, 42].

Interestingly, *P. tricornutum* combines histone PTMs found in both mammals and plants, such as acetylation and mono- and di-methylation of lysine 79 of histone H3 found only in human and yeast [44] but not in *Arabidopsis* [23], underlying the mosaic nature of the *P. tricornutum* genome. Another interesting example is the acetylation of lysine 20 of histone H4, which is shared with *Arabidopsis* but different from human where this residue is only methylated [23]. H4K20me, which is known to be a repressive mark, was not detected by either MS or western blotting using an antibody that recognizes this modification in *Arabidopsis* (data not shown). Furthermore, mono- and di-methylation of lysine 79 of histone H4 are modifications that *P. tricornutum* shares only with *Toxoplasma gondii*, which is an obligate intracellular parasitic protozoan belonging to the Alveolata, a superphylum closely related to Stramenopiles. Novel histone core domain modifications identified in *P. tricornutum* are probably ancient modifications that likely were lost from the divergent lineages (or have not yet been detected). It will be interesting to investigate the presence of these novel PTMs in closely related species to trace back their evolutionary history more precisely.

We further generated an integrated epigenomic map of five histone marks known to be activating or repressive using ChIP-Seq. Combined with previously published genome-wide DNA methylation data [14], comprehensive and combinatorial analyses revealed some conserved and specific epigenetic features in *P. tricornutum*, thereby extending the existence of the histone code to Stramenopiles. As expected, both acetylation of lysines 9 and 14 and di-methylation of lysine 4 of histone H3 map predominantly to genes, followed by intergenic regions and TEs. This is in contrast to H3K9me3, H3K9me2 and H3K27me3, which we found mainly within TEs. As in yeast, mammals and plants, H3K4me2 in *P. tricornutum* does not appear to index genes in relation to their expression level and may not be directly implicated in transcriptional activation [45, 46]. By contrast, acetylation correlates with transcription activation and is enriched in gene promoters, which is in line with genome-wide studies in yeast, human and *Arabidopsis* [47–49]. H3K9me2 marking was mainly found on TEs and a substantial number of transcriptionally repressed genes, consistent with what has been observed in plants and animals in which this mark has been profiled [33, 49–50]. The H3K9me3 mark mapped mainly on TEs and is repressive, which is similar to mammals but different from *Arabidopsis*, where it is exclusively found on euchromatic regions where it has a positive effect on gene regulation [32].

H3K27me3 covered 3.84 Mb (14 %) of the 27.4 Mb *P. tricornutum* genome, which is more than *Neurospora*, *Arabidopsis*, *Drosophila* and mammals, where it covers around 6 % of each genome [25, 32]. This high percentage compared with other species can be explained by the large coverage of H3K27me3 over TEs and is in line with this mark being an ancient histone modification with a primary role in TE silencing, as previously suggested [25]. H3K27me3 is known to be repressive and to mark mainly genes in *Drosophila*, mammals, *Arabidopsis* and *Neurospora* [25, 32]. However, its distribution is different and unusual in *P. tricornutum*, being predominantly on TEs, and it has a repressive effect, implying that the functions and mechanisms of H3K27me3 in single-celled eukaryotes may be different from in their multicellular counterparts.

The H3K27me3 mark is established by the Polycomb repressive complex PRC2 and its absence in the model unicellular fungi *S. pombe* and *S. cerevisiae* initially suggested that it arose to regulate developmental processes in multicellular organisms [53]. This hypothesis has recently been questioned because PRC2 has been found in several single-celled species [37]. Our results showing genome-wide mapping of H3K27me3 in a unicellular organism confirm its early evolution prior to the last common ancestor of animals and plants.

Unlike in *Arabidopsis*, where H3K27me3 marks short regions, typically <1 kb, which tends to be restricted to the coding regions of single genes, H3K27me3-modified regions show blanket-type coverage over large domains in *P. tricornutum* (≥2 kb), which resembles the enriched profiles of H3K27me3 in animals [35, 53]. Our observation of H3K27me3 enrichment over promoter regions and its correlation with highly transcribed genes is also surprising and contrasts with what has been previously reported [37, 55]. The blanket-like coverage of H3K27me3 in animals has been overlooked and such correlations might have been missed from *Drosophila*, human and mouse cells because the wide enrichment of H3K27me3 does not appear to have been analyzed in detail. However, a more detailed study reported recently in mouse embryonic stem cells revealed a similar profile, where H3K27me3 mapping on promoters correlated with high expression, suggesting that these regions might serve as bivalent domains harboring additional activation marks such as H3K4me3 and H3K36me3 [30]. The observed enrichment over the entire gene, gene body alone, or together with either 500 bp upstream or downstream regions represents the majority of H3K27me3-marked genes in *P. tricornutum* and correlates with low expression, thus corresponding to the canonical view of H3K27me3 as being inhibitory to transcription [25, 31, 35]. This suggests that a repressive role of H3K27me3 in *P. tricornutum* might be mediated by gene body marking that occurs in all four clusters identified in Fig. 4, and may compromise transcription elongation. This does not exclude repression by transcription initiation for the upstream marked regions. A novel and intriguing pattern is the presence of H3K27me3 downstream of gene bodies that correlates with activation, suggesting that H3K27me3 does not interfere with transcription termination and that other unknown additional factors allow the transcription of these genes to take place. Overall, H3K27me3-marked genes belong to many functional categories. However, there is a tendency for H3K27me3 to mark genes that have no known function or to be poorly conserved, among which a large proportion have no orthologs. For the rest of the genes that are functionally annotated, a significant number encode ‘developmental’ genes, as seen in mammals and *Arabidopsis*. Cluster-wise, most of the genes marked at their promoter by H3K27me3 are co-marked by acetylation, which might explain their transcriptional activity, while the others, in particular those marked over their entire length, tend to encode ‘developmental’ genes as well as defense response genes.

Our work has also shown the importance of chromatin level regulation in diatoms in response to nitrate starvation, as the changes in the examined histone marks had a considerable impact on gene expression. Epigenetic profiling of nitrate-starved cells revealed a set of genes involved in nitrate assimilation, transport and metabolism which either gain activating marks or lose repressive marks and become upregulated. As expected, many diatom-specific genes of unknown function show up in this analysis, suggesting a key role in surviving nitrate starvation. These uncharacterized genes might help diatoms to cope with a scarcity of nitrate until better conditions become available and allow them to bloom and out-compete other plankton. Functional characterization of these genes will shed light on the pathways that diatoms recruit to survive nutrient depletion and will ultimately contribute to better understanding of diatom ecological success in contemporary oceans.

In line with previous studies in *Drosophila* and *Arabidopsis*, where different chromatin states have been identified, the combinatorial analysis of histone marks with DNA methylation allowed us to define three chromatin states — active, repressive or intermediate — supporting the existence of an epigenetic code in addition to the histone code in *P. tricornutum*. Mapping of additional marks will undoubtedly refine this analysis and provide new insights into the role of chromatin modifications in marine diatoms.

## Conclusions

To gain insights into the evolution of chromatin-mediated regulation of genes, we used an integrative approach combining MS, ChIP and RNA-Seq to analyze post-translational modifications of histones in a stramenopile, the model diatom *P. tricornutum*, which is phylogenetically distant from well-known model organisms from other lineages of life such as plants and animals. MS analysis revealed the strong conservation of histone modifications across distantly related species but also new ones, thus expanding the list of histone PTMs in eukaryotes. Remarkably, *Phaeodactylum* combines histone PTMs found in plants and/or mammals, underscoring the chimeric nature of its genome and suggesting a different evolution of histone PTMs in plants and animals. Genome-wide mapping of some key PTMs revealed shared features with plants and animals, such as the distribution of acetylation, and di-methylation of lysine 4 of histone H3, which map mainly on genes and have an activating effect. Our work shows also some divergence from green lineages exemplified by the H3K9me3 profile, which is found exclusively on genes and is activating in *Arabidopsis* while it is distributed mainly on TEs and is repressive in *P. tricornutum* and animals. Interestingly, the pioneering genome-wide mapping of H3K27me3 has revealed an unorthodox distribution as it maps mainly on TEs and has a repressive effect, while this mark is known to repress mostly genes in euchromatic regions in *Arabidopsis*. The H3K27me3 profile in *P. tricornutum* suggests this mark has an evolutionarily ancient function in transcriptional repression of TEs. The presence of H3K27me3 in *P. tricornutum* and several other algae suggests an ancient origin of Polycomb repressive complex proteins and raises the question of its role in single-celled species. Combinatorial analysis of histone PTMs revealed different chromatin states and gene expression patterns, extending the histone code to Stramenopiles. Investigation of histone modifications under nitrate-limiting conditions revealed the dynamic role of chromatin modifications in regulating some key target genes, indicating their importance for adaptation of diatoms to changing environments.

## Materials and methods

### Materials and growth conditions

*Phaeodactylum tricornutum* Bohlin Clone Pt1 8.6 (CCMP2561) cells were grown as described previously [54]. Under low nitrate, cells were grown as described in [14].

### Extraction of histones

Histones from *P. tricornutum* were extracted as described previously [20].

### MNase digest assay

MNase digest was performed as described previously [54] with a few modifications. Nuclei were washed three times with MNase digestion buffer. The nuclei suspension was aliquoted into 100 μl to which 0.5, 12 and 16 units of MNase were added. After 1 h of incubation with the stop buffer, 1 μl of RNAse was added to each sample and further incubated as described previously [55].

### MS assay

#### Protein in-gel digestion using multiple proteases

Comprehensive localization of PTMs on histones requires observation of each amino acid. Efforts to increase histone coverage have been achieved by use of a multiple protease strategy and chemical derivatization. Enzymatic digestion with trypsin results in small peptides that are difficult to retain on nano-high-performance liquid chromatography (HPLC) columns for analysis by MS. As an alternative, lysine amino groups can first be chemically modified by reaction with propionic anhydride to further generate propionylated residues that would be resistant to trypsin proteolysis. Under these conditions, reproducible and MS-compatible Arg-C-type peptides can be obtained [56].

Proteins were separated by 14 % SDS-PAGE gels and stained with colloidal Coomassie blue (LabSafe Gel Blue™, AGRO-BIO) reagent, which does not contain methanol or acetic acid. Histone bands were excised and washed and proteins were reduced with 10 mM dithiothreitol prior to alkylation with 55 mM iodoacetamide or chloroacetamide for ubiquitylation studies. After washing and shrinking of the gel pieces with 100 % acetonitrile, propionylation or in-gel digestion was performed. All digestions were performed overnight in 25 mM ammonium bicarbonate at 30 °C, by adding 10–20 μl endoproteinase (12.5 ng/μl) trypsin (Promega) or 12.5 ng/μl chymotrypsin (Promega) or 12.5 ng/μl ArgC (Promega) or 20 ng/μl elastase (Sigma-Aldrich). The shrunken gel bands were chemically derivatized by treatment with propionic anhydride before and after trypsin digestion. Briefly, this reaction mixture was created using 3/4 propionyl anhydride (Sigma-Aldrich) and 1/4 methanol. Propionylation reagent (20 μl) and 100 μl of 25 mM ammonium bicarbonate were added to each band, adjusted to pH 8.0, and allowed to react at 51 °C for 20 minutes and reduced to dryness using a SpeedVac concentrator for removal of reaction remnants before trypsin digestion. A second round of propionylation was performed to propionylate the newly created peptide N-termini. Ultrasound-assisted extraction was used to extract peptides with 60 % acetonitrile/5 % formic acid extraction solution. The extract was dried in a vacuum concentrator at room temperature and re-dissolved in solvent A (2 % acetonitrile, 0.1 % formic acid). Peptides were then subjected to MS analysis.

### MS and data analysis

Samples were analyzed by nano-HPLC/MS/MS using an Ultimate3000 system (Dionex S.A.) coupled to an LTQ-Orbitrap mass spectrometer (Thermo Fisher Scientific, Bremen, Germany). Samples were loaded on a C18 pre-column (300 μm inner diameter × 5 mm; Dionex) at 20 μl/minute in 2 % acetonitrile, 0.1 % trifluoroacetic acid. After 3 minutes of desalting, the pre-column was switched on line with the analytical C18 column (75 μm inner diameter × 50 cm; C18 PepMap™, Dionex) equilibrated in 100 % solvent A. Bound peptides were eluted using a 0 to 30 % gradient of solvent B (80 % acetonitrile, 0.085 % formic acid) during 157 minutes, then a 30 to 50 % gradient of solvent B during 20 minutes at a 150 nl/minute flow rate (40 °C). Data-dependent acquisition was performed on the LTQ-Orbitrap mass spectrometer in the positive ion mode. Survey MS scans were acquired on the Orbitrap in the 400–1200 m/z range with resolution set to a value of 100,000. Each scan was recalibrated in real time by co-injecting an internal standard from ambient air into the C-trap (‘lock mass option’). The five most intense ions per survey scan were selected for collision-induced dissociation fragmentation and the resulting fragments were analyzed in the linear trap (LTQ). Target ions already selected for MS/MS were dynamically excluded for 20 s.

Data were acquired using the Xcalibur software (version 2.0.7) and the resulting spectra were then analyzed via the Mascot™ Software created with Proteome Discoverer (version 1.4, Thermo Scientific) using an in-house database containing the sequences of histone proteins from *P. tricornutum* (PtH3_50695, PtH3_21239, PtH4_26896, PtH2A_34798, PtH2A_28445, PtH2B_11823, PtH1_54381) or the UniProtKB Phaeodactylum tricornutum database (15,832 proteins) with a Mascot score of 1 % FDR (or <5 %; shown in bold in Additional file 16). Carbamidomethylation of cysteine, oxidation of methionine, acetylation of lysine and protein N-termini, methylation, dimethylation of lysine, arginine and trimethylation of lysine, methylation of aspartic and glutamic acid, di-glycine of lysine, propionylation of lysine and N-termini of peptides, phosphorylated histidine, serine, threonine and tyrosine were set as variable modifications for Mascot searches. The mass tolerances in MS and MS/MS were set to 5 ppm and 0.5 Da, respectively. The resulting Mascot files were further processed using myProMS [57]. The mass spectrometry proteomics data have been deposited to the ProteomeXchange Consortium [58] via the PRIDE partner repository [59] with the dataset identifier PXD002148.

### Isolation and immunoprecipitation of chromatin

Chromatin isolation and immunoprecipitation were performed as described previously [21]. The following antibodies were used for immunoprecipitation: H4K9/14Ac (005–044) from Diagenode; H3K4m2 (32356) and H3K9me3 (8898) from Abcam; H3 (07–690), H3K4me2 (07–030), H3K9me2 (17–681) and H3K27me3 (07–449) from Millipore. Peptide competition assays were performed for H3K4me2, H3K9me2 and H3K27me3 as described previously [21].

### Chlorophyll analysis

Cells (1 ml) were harvested by centrifugation at 1400 *g* for 10 minutes, resuspended in 100 % ethanol, and incubated for 30 minutes in the dark. The crude extract was cleared by 1-minute centrifugation at 12,000 *g* and the supernatant was used for chlorophyll quantification according to [60] with a spectrophotometer Biossacte 3 from Thermo Spectronic reading at 629 nm and 665 nm wavelengths. The calculated chlorophyll contents were normalized to 10^6^ cells.

### Oxygen evolution

Photosynthesis was measured as O_2_ exchange rates using a Clark-type oxygen electrode at 25 °C (Oxy-Lab, Hansatech Instruments, King’s Lynn, UK). The actinic light was provided by light-emitting diodes with an emission maximum around 650 nm. For each measurement cells were concentrated by 10-minute centrifugation at 1400 *g* and resuspended in Artificial Sea Water (ASW) to a final concentration of 10^7^ to 3 × 10^7^ cells/ml. Net O_2_ evolution V_max_ was measured at 800 μE and is presented as nmol O_2_ evolved per minute per 10^6^ cells.

### Data analysis

For mapping and analysis we used *P. tricornutum* genome v.2.0 available at the Joint Genome Institute [61]. Reads obtained were quality controlled with a standardized procedure using FASTQC [62]. Trimmomatic [63] was used for quality trimming. GO-based functional analysis on ChIP-marked genes and methylated genes were performed using BLAST2GO [64] with a significant FDR cutoff of 0.05 % probability level. R [65] and Biopython [66] were extensively used for data analysis. For pattern-based analysis on genes and flanking regions, genes were normalized to equal size, and flanking 2-kb regions were selected as the average intergenic size of ~1500 bp. Data processing, analysis, and plotting were performed using Python, R/Bioconductor and Hyperbrowser [67]. Results of the analysis have been made available on the Gbrowse-based genome browser at [68].

### Computational analysis of histone modifications in *P. tricornutum* by ChIP-Seq

Single-end sequencing of the five ChIP samples was performed using an Illumina GAIIx with a read length between 36 and 50 bp. This yielded an average of approximately 37 million reads each (Additional file 3). Data for all ChIP samples and input were of good quality with mean quality scores of 30, with 50 % mean GC content. The reads were mapped onto the *P. tricornutum* genome v.2.0 using Bowtie [69] with mismatch permission of 2 bp. Unique mapping of reads was adopted. To identify regions that were significantly enriched, we used MACS [70] and SICER [71] with parameters of W:200 (window length), G:200 (gap size) for H3K4me2 and H3K9_14Ac; W:200, G:600 for H3K27me3, H3K9me2 and H3K9me3; and a FDR <1E-2. Enriched regions were detected against the islands of background control with the same parameter.

MACS and SICER both generated peaks with similar peak ranges and comparable overlapping genomic regions. But for H3K9me2 and H3K27me3, MACS showed much fewer peaks and in these two cases SICER showed significant diffused peaks which overlapped on repeat regions. Visualization and analysis of genome-wide enrichment profiles were done with IGV [72]. Peak annotations such as proximity to genes and overlap on genomic features such as transposons and genes were performed using Peak Analyzer [73]. The high-throughput Chip sequencing data have been deposited in NCBI’s Gene Expression Omnibus under accession number GSE68513.

### H3K27me3 clustering

A 700 × 30 matrix was created based on position of the H3K27me3 mark along the 500 bp upstream, gene body, and 500 bp downstream regions. Hierarchical clustering was done on this matrix using the complete-linkage method [74]. This resulted in six clusters and each cluster was then correlated to expression level. Both expression levels and ChIP seq peaks were plotted as heat maps with red reflecting high expression (expression level RPKM greater than 20), and green low expression (expression level RPKM between 0 and 20).

### Homology estimation analysis

We downloaded 57,224,756 protein sequences from 344,297 species from UniProt [75] and MMETSP [76] databases. The sequences were then categorized into various groups of life, Archaea, Bacteria and as described in the Eukaryote tree of life [77]. Finally, for the homology estimation analysis, 662,593, 20,176,346, 7,250,814, 1,741,270 and 3,065,341 protein sequences from Archaea (~267 species), Bacteria (~4435 species), Opisthokonta (~853 species), Archaeplastida (~144 species) and diatoms (~70 species) were considered, respectively. The homology was assigned to a pair by BLASTP [78] using an expected cutoff value of 1e-5.

### Detection of nucleosome occupancy sites

Nucleosome prediction with NuPoP [79] (HMM order, 3; Markov model, Linker-Nucleosome; Markov model species, NULL) on the Pt1.86 were compared with nucleosome mapping using NucHunter [80]. The following parameters are used: chunk size, 1 Mb; *p*-value threshold, 1E-6; Z-score, 3.0; interval length, 146 bp. NuPoP predicts 161,875 nucleosomes. Genome-wide di-nucleotide preference over nucleosomes and nucleosome-depleted regions was estimated by fetching their corresponding nucleotide sequences using GFF-Ex [81] and calculating the frequency of AT, TA, TT, GC and CG occurrence within the sequences using compseq [82].

### Bisulfite-Seq methylation analysis

We mapped Bisulfite-Seq reads from an Illumina GAII from DNA extracted from both nitrate replete and depleted conditions after filtering through FASTQC to the Pt1.86 reference genome available using Bismark [83]. Five million reads for the replete nitrogen condition and 3.3 million reads for the low nitrogen condition were uniquely mapped and de-duplicated. Average fold coverage was 17. We extracted the methylation calls for each base and for calling a CpG/CHH/CHG site as methylated, we used a cutoff of at least three reads and a minimum of 20 % reads being methylated.

### RNA-Seq data analysis for gene expression quantification

TopHat v.1.1.3 [84] and Cufflinks [85] were used to map and estimate the transcripts from the RNA-Seq data. Relative abundances of transcripts were measured as fragments per kilobase of exon per million fragments mapped (FPKM).

## Additional files

Additional file 1: Figure S1. Phylogeny of different classes of histone proteins. **A** Relationship between different classes of histone proteins in *P. triconutum*. Boot strap numbers are indicated. **B** Protein sequences from different species belonging to respective classes of histone proteins were obtained from NCBI, aligned using Gonnet [87], and used to construct a neighbor-joining tree. The numeric values on the tree indicate the bootstrap confidence between two branches. Only core histones are considered in this tree. NCBI accession numbers used are as follows: [H3: *Drosophila melanogaster* (NP_724345), *Homo sapiens* (NP_003520), *Caenorhabditis elegans* (NP_496899), *Arabidopsis thaliana* (NP_195713), *Chlamydomonas reinhardtii* (XP_001690671), *Phaeodactylum tricornutum* (XP_002177514), *Thalassiosira pseudonana* (XP_002288694), *Paramecium tetraurelia* (BAF03646), *Saccharomyces cerevisiae* (AAS64349), *Dictyostelium discoideum* (XP_647577), *Leishmania infantum* (XP_001463740), *Giardia intestinalis* (ESU45648)]; [H4: *Arabidopsis thaliana* (NP_180441), *Chlamydomonas reinhardtii* (XP_001690685), *Caenorhabditis elegans* (NP_492641), *Drosophila melanogaster* (NP_524352), *Paramecium tetraurelia* (CAD97571), *Dictyostelium discoideum* (XP_642712), *Leishmania infantum* (XP_001464339), *Giardia intestinalis* (ADW95184), *Saccharomyces cerevisiae* (EDV12280), *Homo sapiens* (ESW55528), *Phaeodactylum tricornutum* (XP_002179286), *Thalassiosira pseudonana* (XP_002288196)]; [H2A: *Phaeodactylum tricornutum* (XP_002181345), *Thalassiosira pseudonana* (XP_002286413), *Paramecium tetraurelia* (XP_001433287), *Drosophila melanogaster* (NP_524519), *Caenorhabditis elegans* (NP_001263788), *Homo sapiens* (NP_003507), *Arabidopsis thaliana* (Q90681), *Chlamydomonas reinhardtii* (EDO96006), *Saccharomyces cerevisiae* (CAA81267), *Leishmania infantum* (CAD11891), *Dictyostelium discoideum* (XP_636327), *Giardia intestinalis* (ESU37209)]; [H2B: *Phaeodactylum tricornutum* (XP_002179210), *Thalassiosira pseudonana* (XP_002290856), *Saccharomyces cerevisiae* (CAA81268), *Arabidopsis thaliana* (NP_180440), *Chlamydomonas reinhardtii* (XP_001700194), *Drosophila melanogaster* (NP_724342), *Caenorhabditis elegans* (NP_505464), *Homo sapiens* (NP_003510), *Giardia intestinalis* (ESU39253), *Paramecium tetraurelia* (XP_001428087), *Leishmania infantum* (XP_001470056), *Dictyostelium discoideum* (XP_628972)]; [H1: *Drosophila melanogaster* (NP_724341), *Homo sapiens* (NP_722575), *Chlamydomonas reinhardtii* (XP_001693443), *Arabidopsis thaliana* (AAD20121), *Caenorhabditis elegans* (NP_491678), *Saccharomyces cerevisiae* (NP_015198), *Phaeodactylum tricornutum* (XP_002179285), *Thalassiosira pseudonana* (XP_002294007)]. Additional file 2: Figure S2. Identification of histone modification sites. MS/MS spectrum of novel PTMs detected in histones H3, H4, H2A and H2B, as well as histone PTMs that were mapped. Additional file 3: Table S1. Sequencing data from each chromatin immunoprecipitation experiment followed by Illumina HiSeq 2000. The number of sequencing reads analyzed in the ChIP-Seq and RNA-Seq data are shown. Additional file 4: Figure S3. Coverage (in base pairs) of the peaks of each histone mark on the genomic features (genes, TEs and intergenice regions) of *P. tricornutum*. Additional file 5: Figure S4. Distributions of histone modifications on genes, TEs, Genes and TEs, and intergenic regions. Additional file 6: Table S2. Functions of encoded proteins of genes marked with a range of histone modifications. KOG functional categories and GO enriched categories are shown. For functional enrichment, a hypergeometric test was performed with KOG classes and a GO enrichment test with GO classes. GO enrichment was performed with a Fisher exact test using a reference set of whole genome annotation (4772 genes) and an FDR value of 0.05. The comparison was performed against the unmarked genes. Additional file 7: Figure S5. Distribution of histone marks on TEs and their correlation with expression. **A** Proportion of different classes of TEs marked by six modifications, including DNA methylation. The numbers of the different classes of TEs are indicated above each bar. **B** Expression of different classes of TEs marked by the five histone modifications. Additional file 8: Figure S6. Nucleosome features. **A** Micrococcal nuclease digest. The three right most lanes indicate digestions of nuclei with increasing concentrations of MNase resulting in a major band of mononucleosomes around 150 bp. Lane C indicates undigested control. The two left-most lanes indicate low and high molecular weight DNA ladders. **B** Pie chart showing genome-wide nucleosome distributions (H3) along genes, TEs, and intergenic regions. The number of each genomic feature is indicated. **C** Dinucleotide frequencies around nucleosome occupancy sites and over linker regions. Additional file 9: Table S3. GO categories and orthology groups of H3K27me3 clusters of genes. Additional file 10: Figure S7. Characterization of *P. tricornutum* cells grown under nitrate limiting conditions. **A** Chlorotic phenotype of cells grown under low nitrate at 75 μM (left) versus replete control at 880 μM (right). **B** Growth curves of cells grown under low (75 μM) and normal (880 μM) nitrate for 3 weeks. **C** Chlorophyll *a* and *c* contents in each culture condition measured using ethanol extraction. **D** Maximal oxygen evolution rates in each culture condition. Measurements in C and D were made after seven days of culture. Additional file 11: Table S4. List of differentially marked and expressed genes under nitrate depleted condition. Additional file 12: Figure S8. GO categories of genes differentially regulated and marked by H3K4me2 under low nitrate. Additional file 13: Figure S9. GO categories of genes differentially regulated and marked by H3AcK9/K14 under low nitrate. Additional file 14: Figure S10. GO categories of genes differentially regulated and marked by H3K9me3 under low nitrate. Additional file 15: Figure S11. MapMan display of genes differentially marked and regulated under low nitrate showing assignment to different metabolic compartments, including light harvesting, photorespiration, amino acid biosynthesis, and lipid metabolism. MapMan of the model plant *Arabidopsis thaliana* was used. Additional file 16: Table S5. The sites of post-tanslational modifications (PTMs) on histones include amino acid (residue) and peptide sequences which are acetylated (*Acetyl*), methylated (*Methyl*, *Dimethyl* and *Trimethyl*) or ubiquitinated.

## Abbreviations

bp: base pair
CDS: coding sequence
ChIP: chromatin immunoprecipitation
CS: chromatin state
FDR: false discovery rate
GO: gene ontology
HPLC: high-performance liquid chromatography
MNase: micrococcal nuclease
MS: mass spectrometry
PTM: post-translational modification
TE: transposable element
