# Supplementary figures and images for: An integrative analysis of post-translational histone modifications in the marine diatom Phaeodactylum tricornutum
Source: Genome Biol. 2015 May 20;16(1):102. doi: 10.1186/s13059-015-0671-8 (PMC4504042; doi:10.1186/s13059-015-0671-8)

**A**

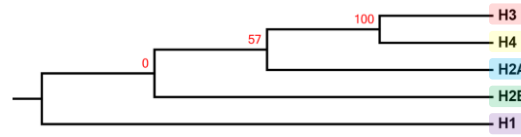

**B**

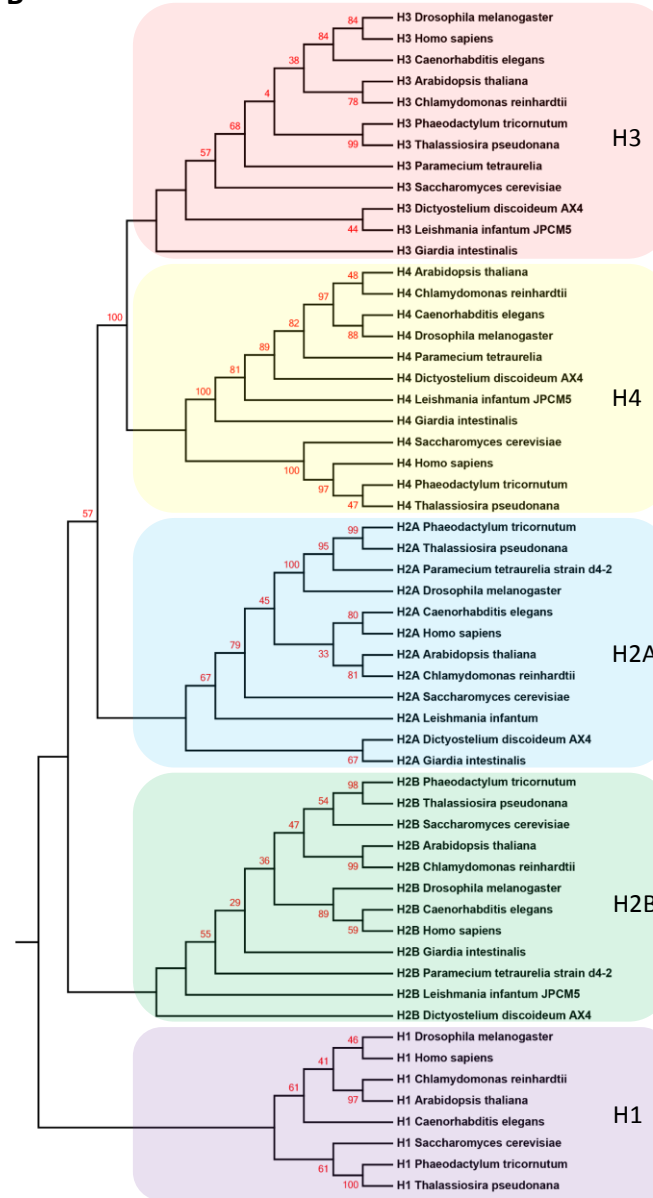

Supplement: Additional file 1: Figure S1. — Phylogeny of different classes of histone proteins. A Relationship between different classes of histone proteins in P. triconutum. Boot strap numbers are indicated. B Protein sequences from different species belonging to respective classes of histone proteins were obtained from NCBI, aligned using Gonnet [87], and used to construct a neighbor-joining tree. The numeric values on the tree indicate the bootstrap confidence between two branches. Only core histones are considered in this tree. NCBI accession numbers used are as follows: [H3: Drosophila melanogaster (NP_724345), Homo sapiens (NP_003520), Caenorhabditis elegans (NP_496899), Arabidopsis thaliana (NP_195713), Chlamydomonas reinhardtii (XP_001690671), Phaeodactylum tricornutum (XP_002177514), Thalassiosira pseudonana (XP_002288694), Paramecium tetraurelia (BAF03646), Saccharomyces cerevisiae (AAS64349), Dictyostelium discoideum (XP_647577), Leishmania infantum (XP_001463740), Giardia intestinalis (ESU45648)]; [H4: Arabidopsis thaliana (NP_180441), Chlamydomonas reinhardtii (XP_001690685), Caenorhabditis elegans (NP_492641), Drosophila melanogaster (NP_524352), Paramecium tetraurelia (CAD97571), Dictyostelium discoideum (XP_642712), Leishmania infantum (XP_001464339), Giardia intestinalis (ADW95184), Saccharomyces cerevisiae (EDV12280), Homo sapiens (ESW55528), Phaeodactylum tricornutum (XP_002179286), Thalassiosira pseudonana (XP_002288196)]; [H2A: Phaeodactylum tricornutum (XP_002181345), Thalassiosira pseudonana (XP_002286413), Paramecium tetraurelia (XP_001433287), Drosophila melanogaster (NP_524519), Caenorhabditis elegans (NP_001263788), Homo sapiens (NP_003507), Arabidopsis thaliana (Q90681), Chlamydomonas reinhardtii (EDO96006), Saccharomyces cerevisiae (CAA81267), Leishmania infantum (CAD11891), Dictyostelium discoideum (XP_636327), Giardia intestinalis (ESU37209)]; [H2B: Phaeodactylum tricornutum (XP_002179210), Thalassiosira pseudonana (XP_002290856), Saccharomyces cerevisiae (CAA81268), Arabidopsis [file 13059_2015_671_MOESM1_ESM.pdf]

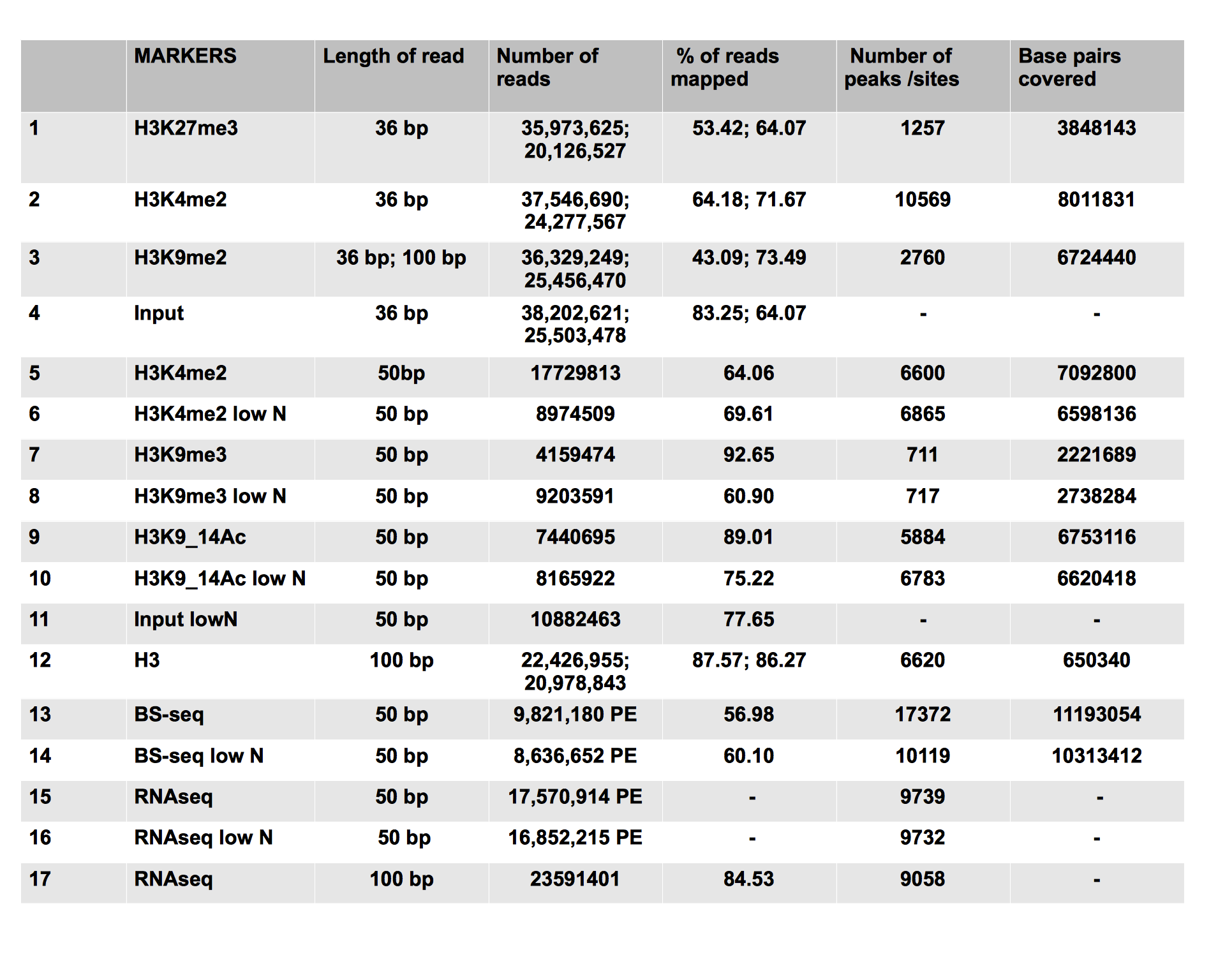

Supplement: Additional file 3: Table S1. — Sequencing data from each chromatin immunoprecipitation experiment followed by Illumina HiSeq 2000. The number of sequencing reads analyzed in the ChIP-Seq and RNA-Seq data are shown. [file 13059_2015_671_MOESM3_ESM.png]

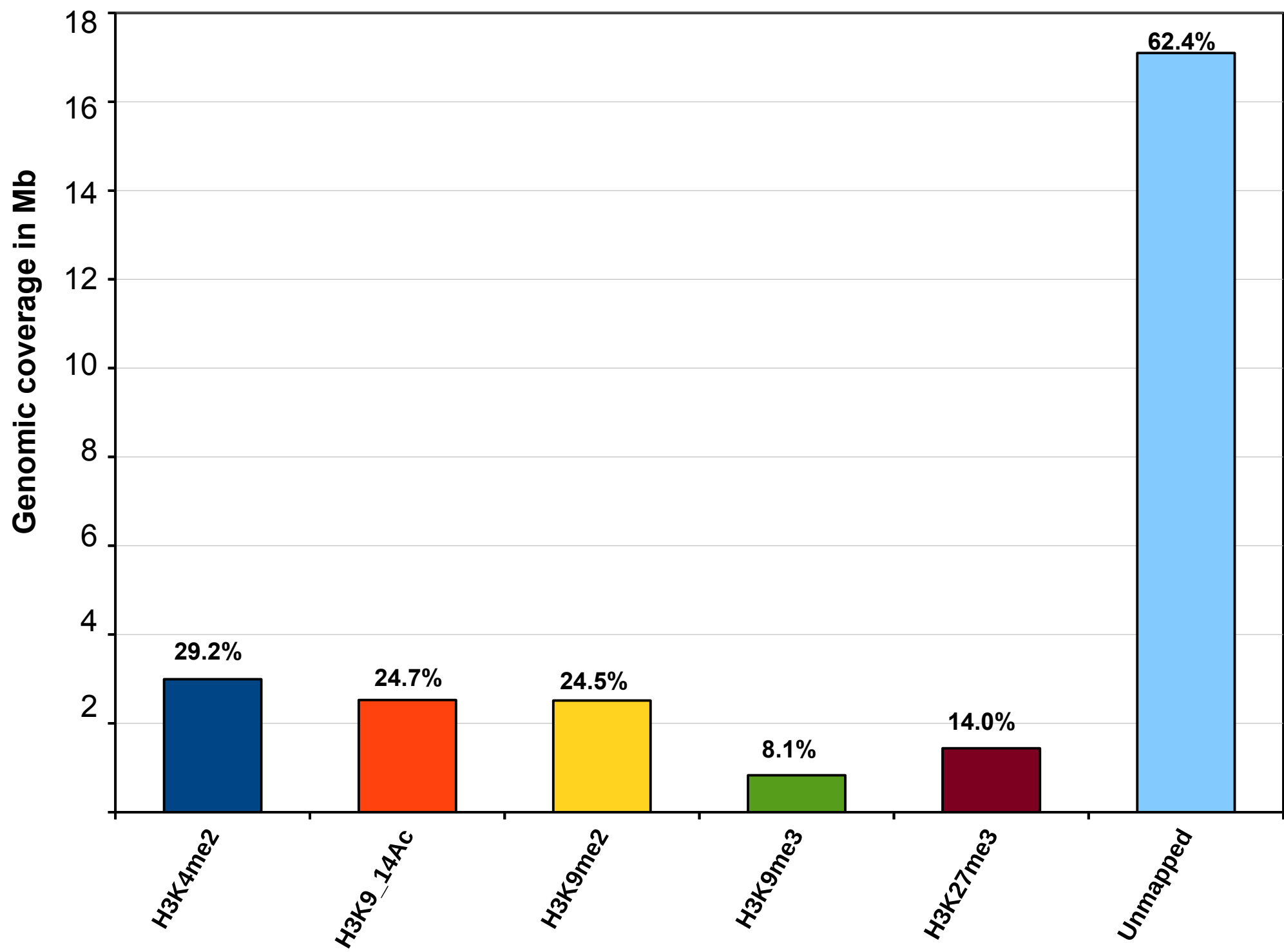

Supplement: Additional file 4: Figure S3. — Coverage (in base pairs) of the peaks of each histone mark on the genomic features (genes, TEs and intergenice regions) of P. tricornutum. [file 13059_2015_671_MOESM4_ESM.pdf]

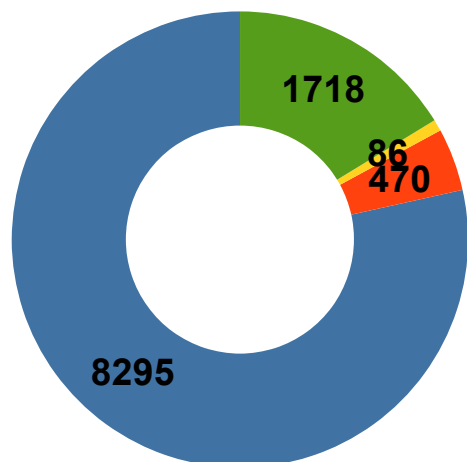

**H3K4me2**

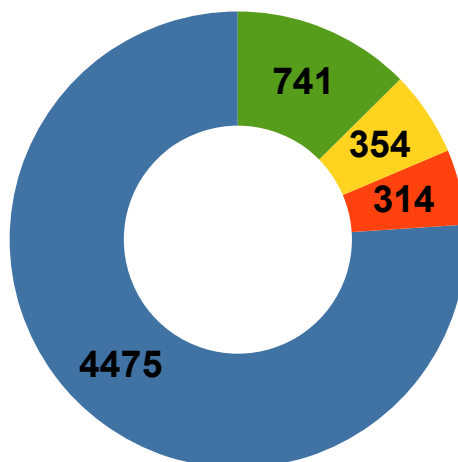

**H3K9\_14Ac**

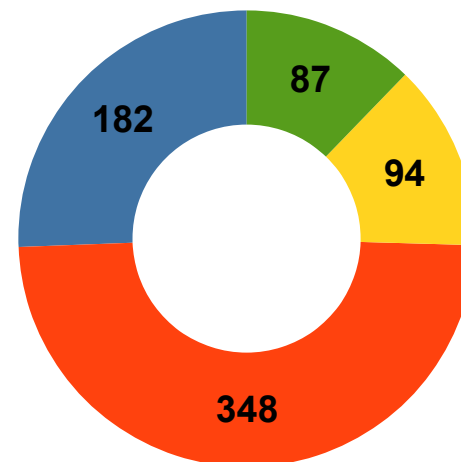

**H3K9me3**

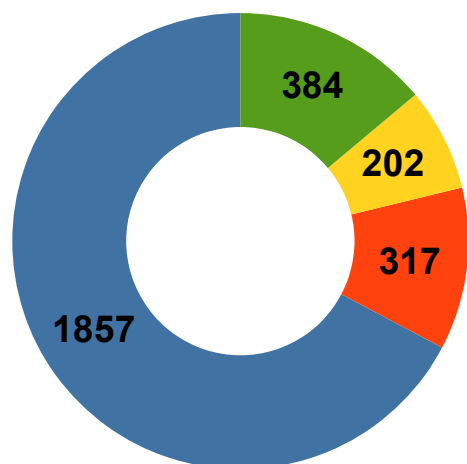

**H3K9me2**

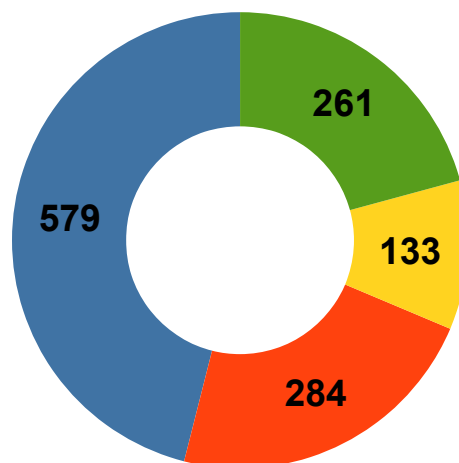

**H3K27me3**

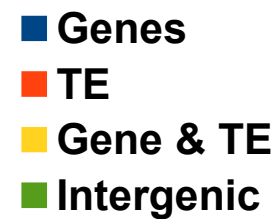

Supplement: Additional file 5: Figure S4. — Distributions of histone modifications on genes, TEs, Genes and TEs, and intergenic regions. [file 13059_2015_671_MOESM5_ESM.pdf]

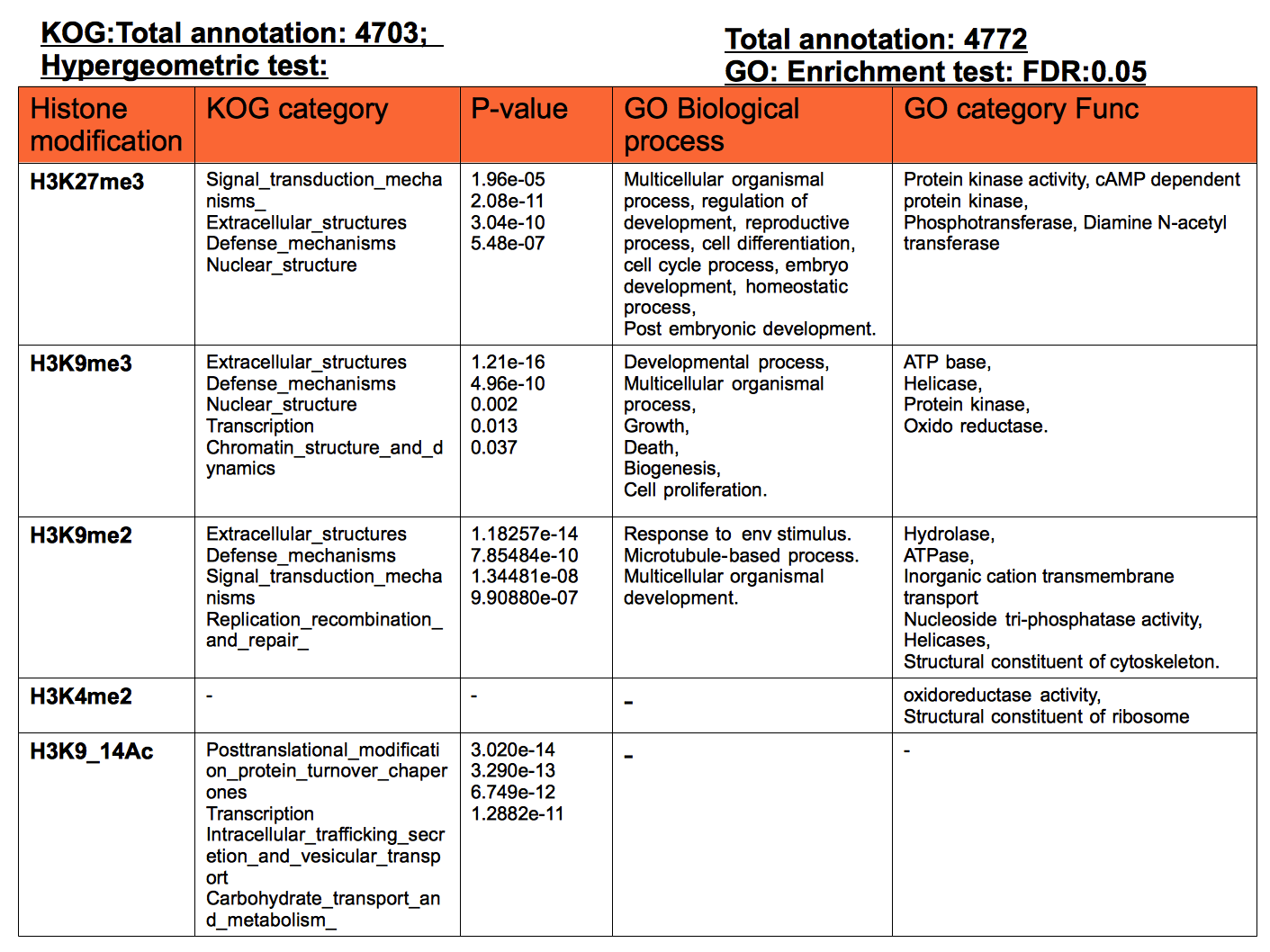

Supplement: Additional file 6: Table S2. — Functions of encoded proteins of genes marked with a range of histone modifications. KOG functional categories and GO enriched categories are shown. For functional enrichment, a hypergeometric test was performed with KOG classes and a GO enrichment test with GO classes. GO enrichment was performed with a Fisher exact test using a reference set of whole genome annotation (4772 genes) and an FDR value of 0.05. The comparison was performed against the unmarked genes. [file 13059_2015_671_MOESM6_ESM.png]

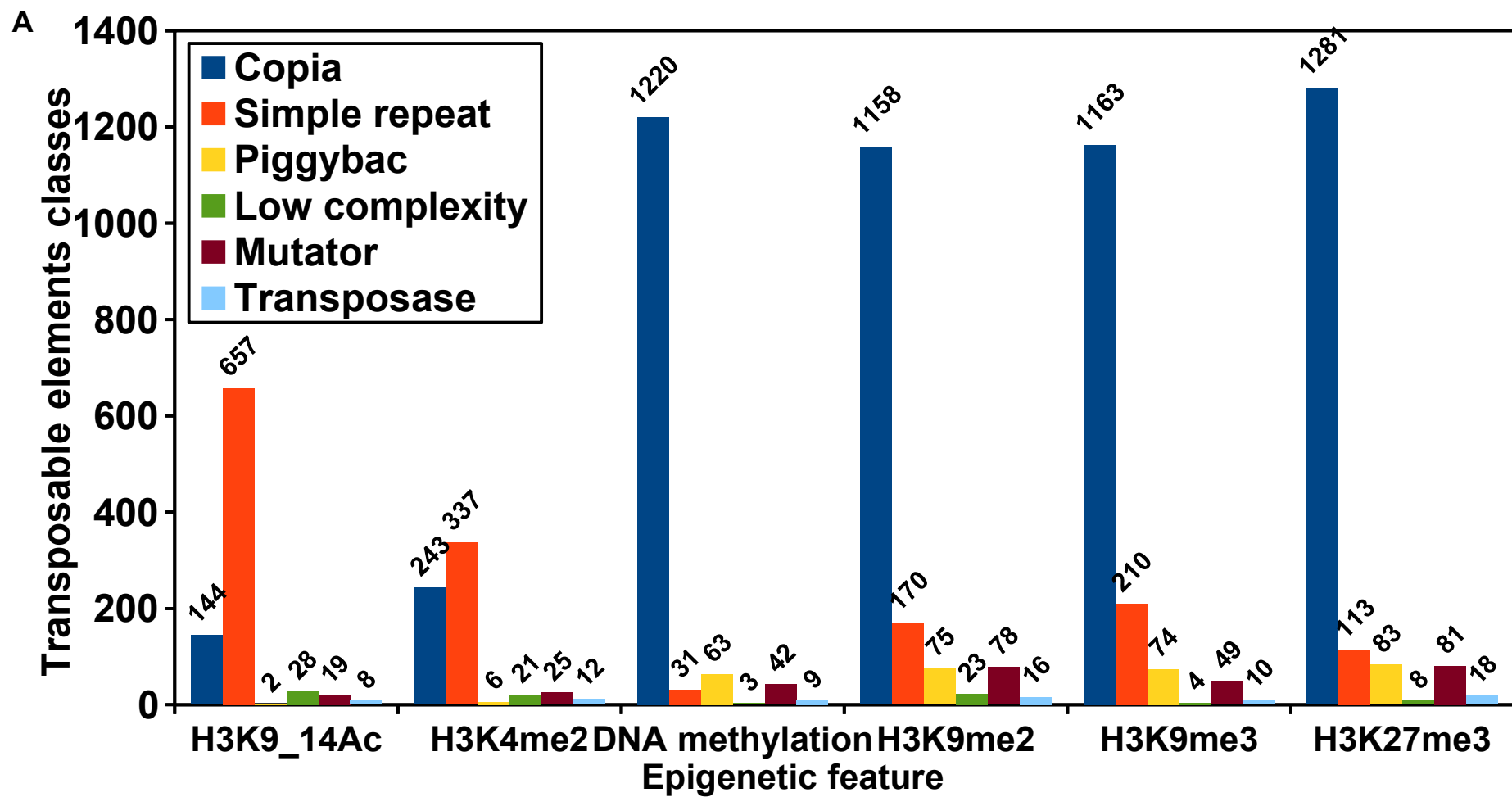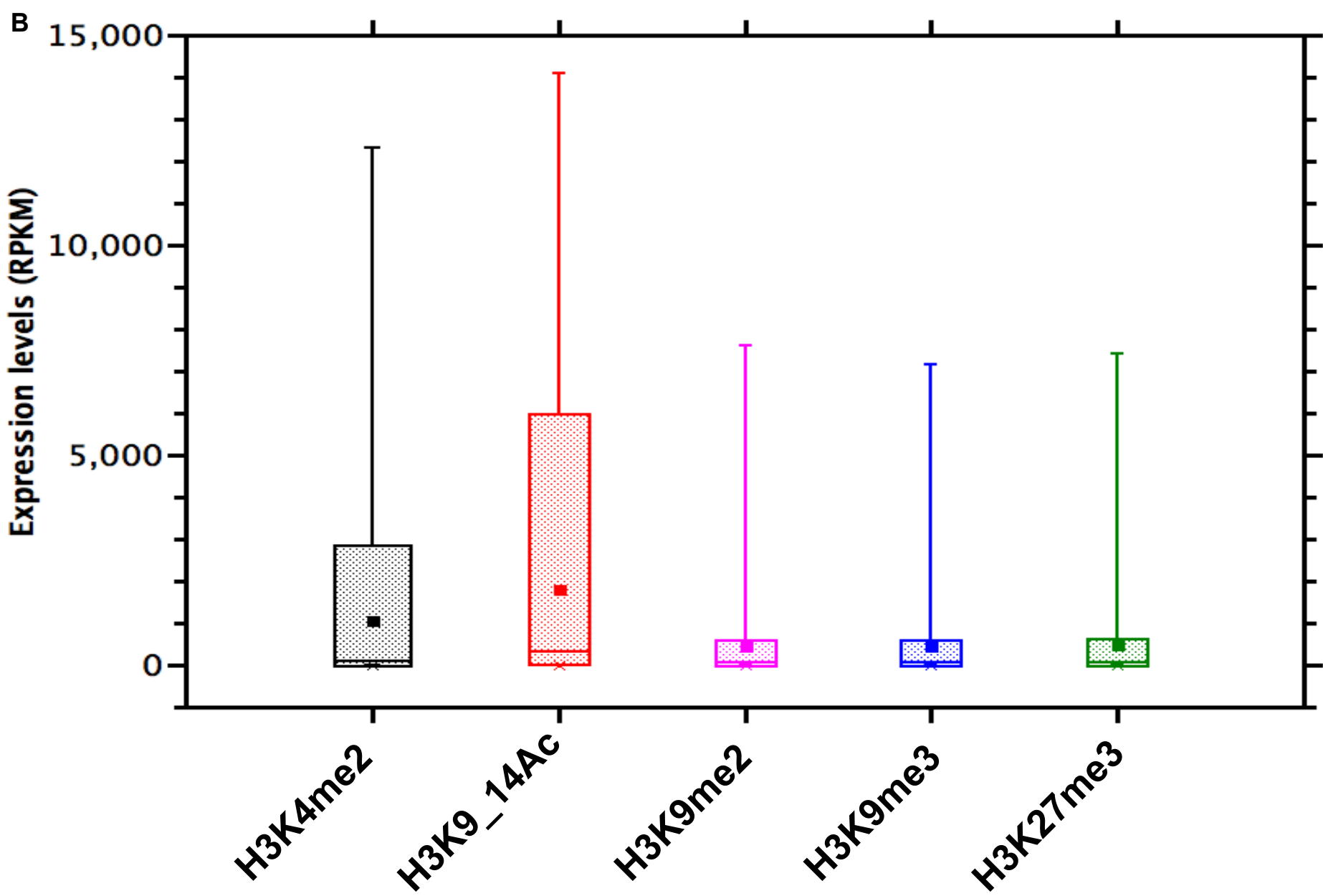

Supplement: Additional file 7: Figure S5. — Distribution of histone marks on TEs and their correlation with expression. A Proportion of different classes of TEs marked by six modifications, including DNA methylation. The numbers of the different classes of TEs are indicated above each bar. B Expression of different classes of TEs marked by the five histone modifications. [file 13059_2015_671_MOESM7_ESM.pdf]

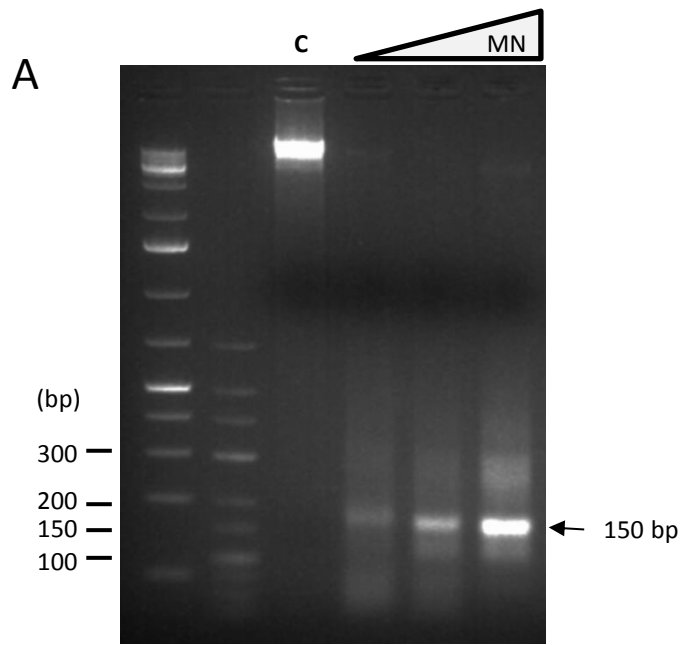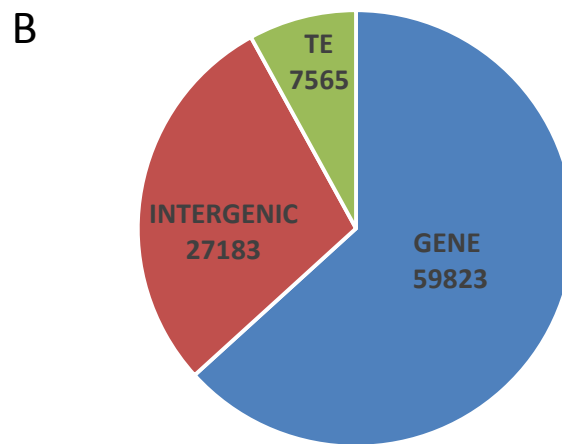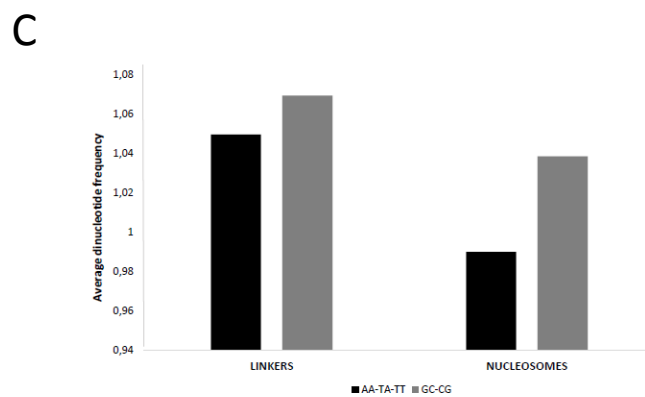

Supplement: Additional file 8: Figure S6. — Nucleosome features. A Micrococcal nuclease digest. The three right most lanes indicate digestions of nuclei with increasing concentrations of MNase resulting in a major band of mononucleosomes around 150 bp. Lane C indicates undigested control. The two left-most lanes indicate low and high molecular weight DNA ladders. B Pie chart showing genome-wide nucleosome distributions (H3) along genes, TEs, and intergenic regions. The number of each genomic feature is indicated. C Dinucleotide frequencies around nucleosome occupancy sites and over linker regions. [file 13059_2015_671_MOESM8_ESM.pdf]

A

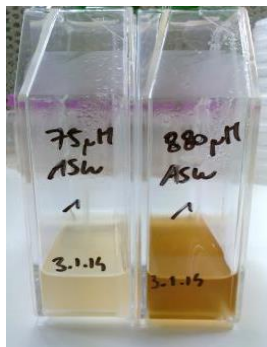

B

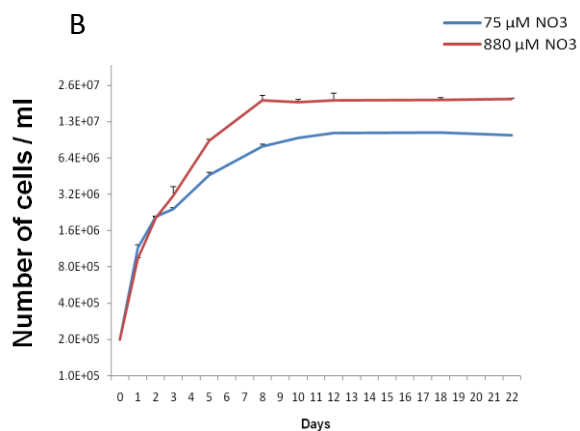

C

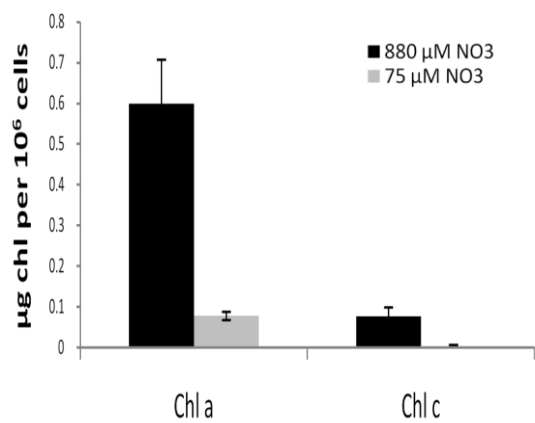

D

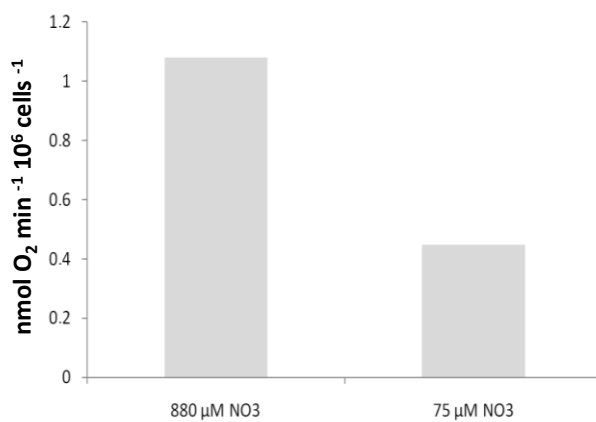

Supplement: Additional file 10: Figure S7. — Characterization of P. tricornutum cells grown under nitrate limiting conditions. A Chlorotic phenotype of cells grown under low nitrate at 75 μM (left) versus replete control at 880 μM (right). B Growth curves of cells grown under low (75 μM) and normal (880 μM) nitrate for 3 weeks. C Chlorophyll a and c contents in each culture condition measured using ethanol extraction. D Maximal oxygen evolution rates in each culture condition. Measurements in C and D were made after seven days of culture. [file 13059_2015_671_MOESM10_ESM.pdf]

# H3K4me2 differentially marked genes

■ NormalN  
■ LowN

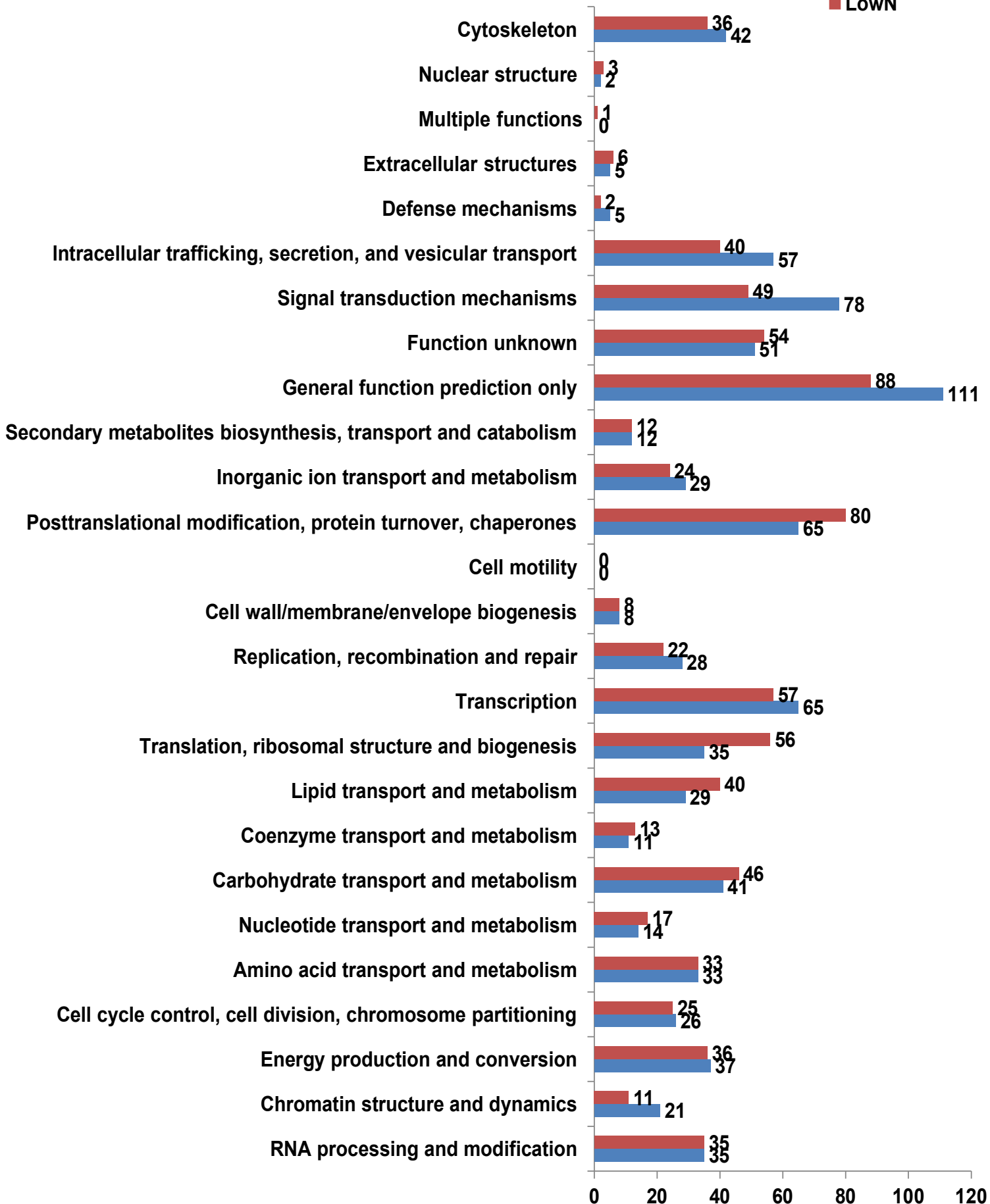

Supplement: Additional file 12: Figure S8. — GO categories of genes differentially regulated and marked by H3K4me2 under low nitrate. [file 13059_2015_671_MOESM12_ESM.pdf]

# H3K9me3 differentially marked genes

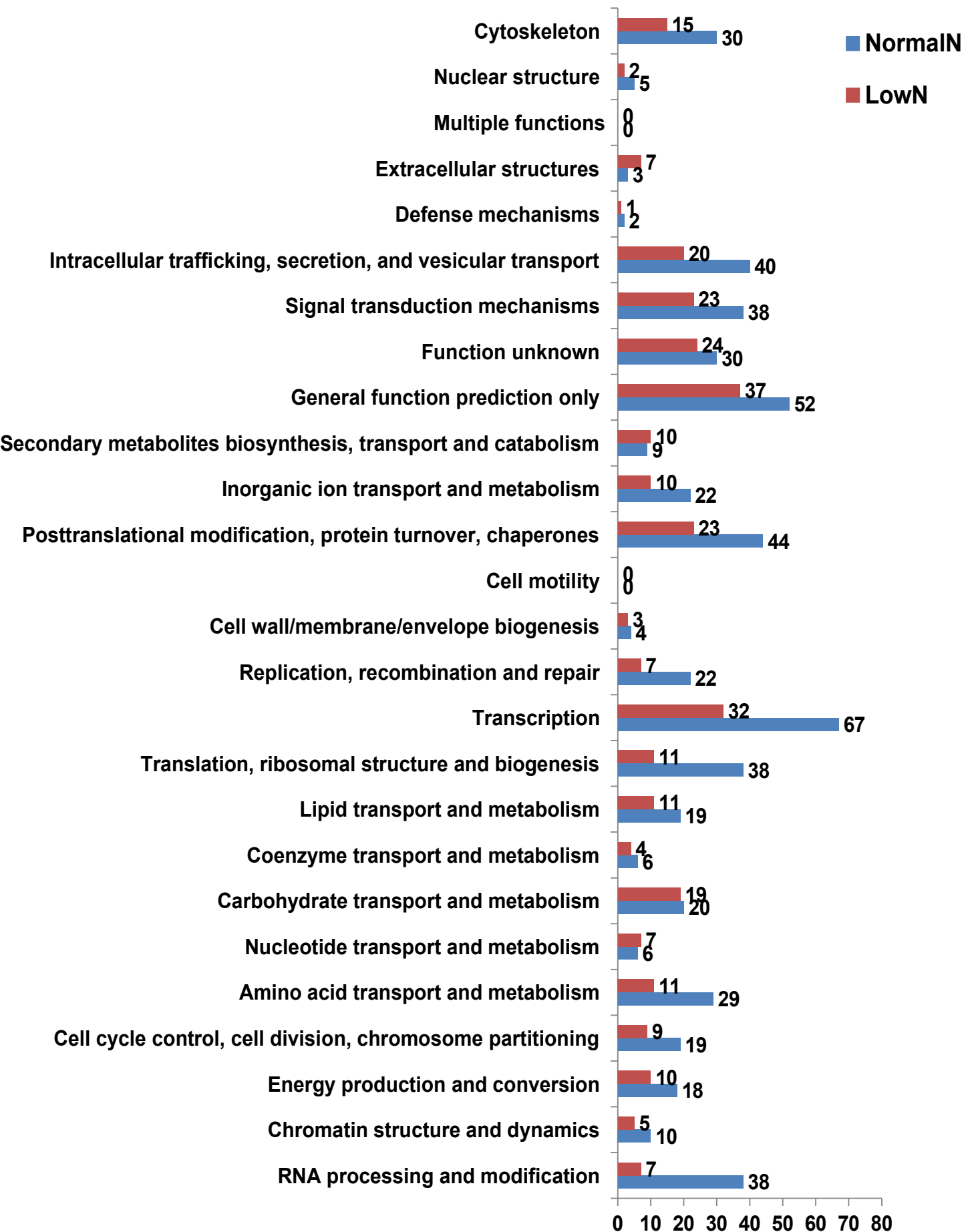

Supplement: Additional file 13: Figure S9. — GO categories of genes differentially regulated and marked by H3AcK9/K14 under low nitrate. [file 13059_2015_671_MOESM13_ESM.pdf]

# H3K9\_14Ac differentially marked genes

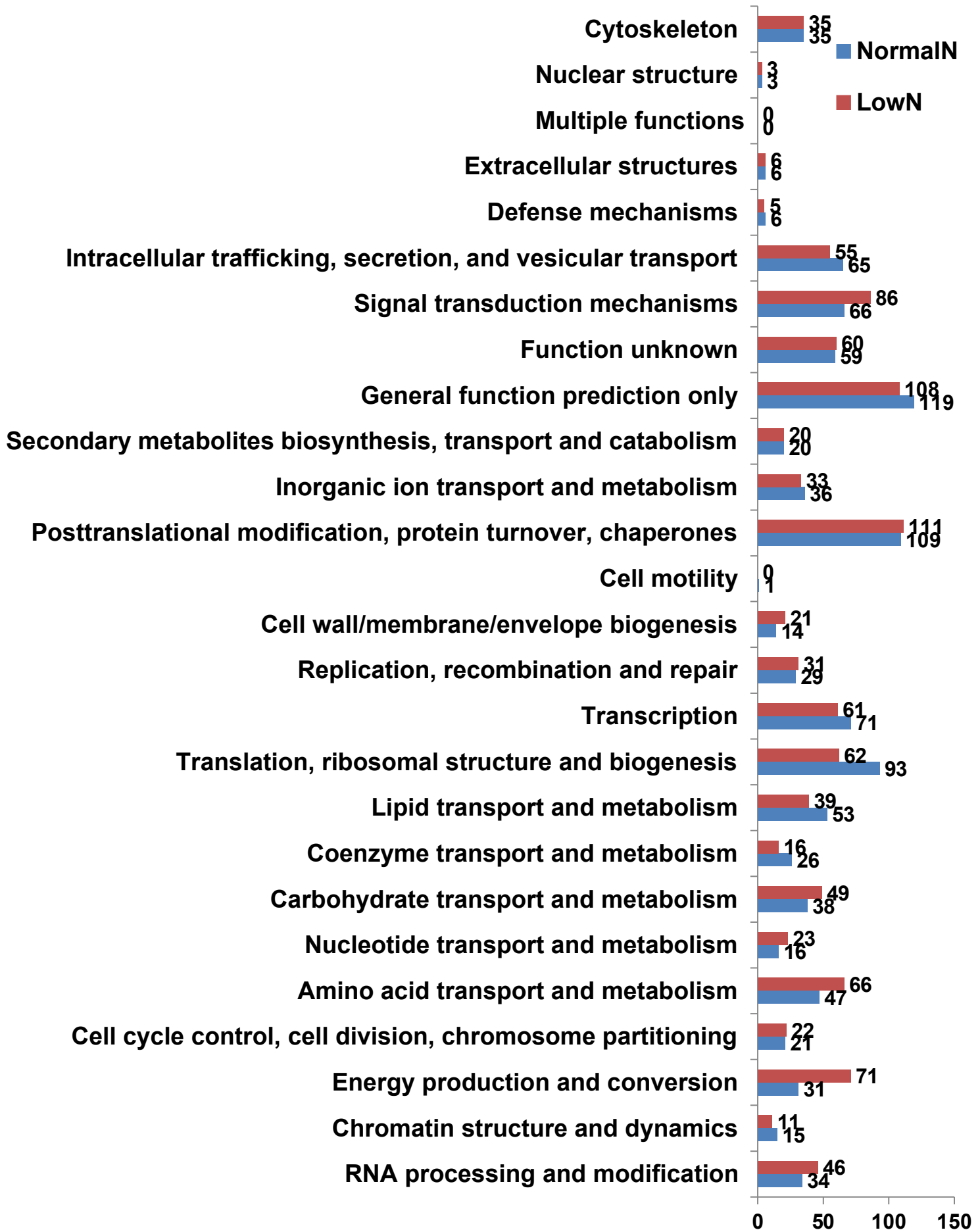

Supplement: Additional file 14: Figure S10. — GO categories of genes differentially regulated and marked by H3K9me3 under low nitrate. [file 13059_2015_671_MOESM14_ESM.pdf]

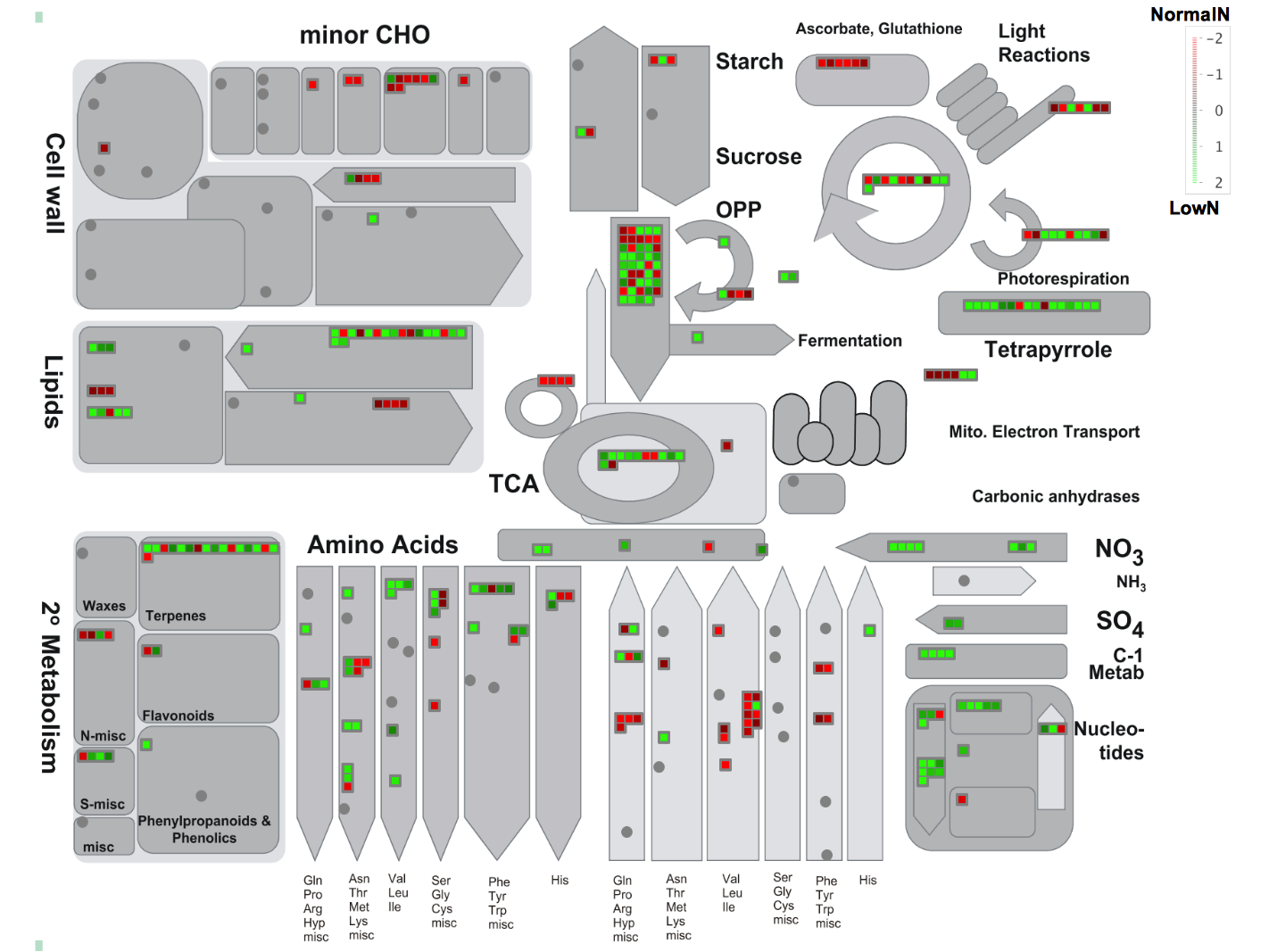

Supplement: Additional file 15: Figure S11. — MapMan display of genes differentially marked and regulated under low nitrate showing assignment to different metabolic compartments, including light harvesting, photorespiration, amino acid biosynthesis, and lipid metabolism. MapMan of the model plant Arabidopsis thaliana was used. [file 13059_2015_671_MOESM15_ESM.png]
