# Supplementary material for: An integrative analysis of post-translational histone modifications in the marine diatom Phaeodactylum tricornutum
Source: Genome Biol. 2015 May 20;16(1):102. doi: 10.1186/s13059-015-0671-8 (PMC4504042; doi:10.1186/s13059-015-0671-8)

H4

Ac at K59

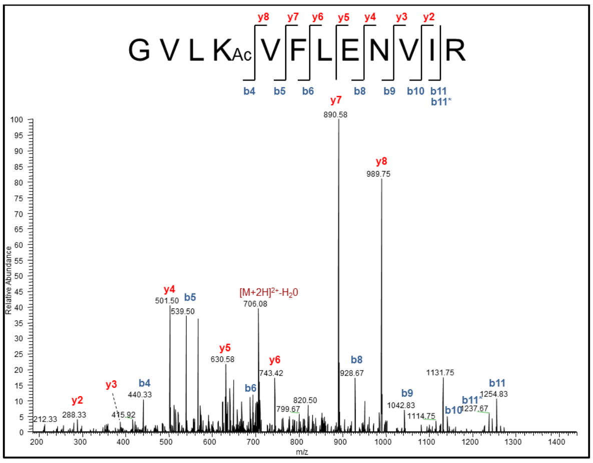

Ac at K31

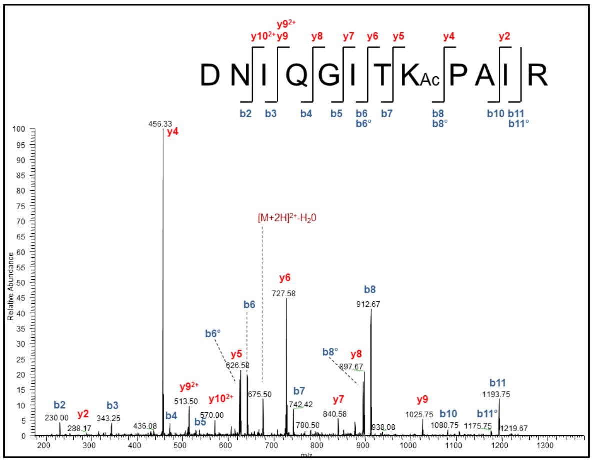

me at K79

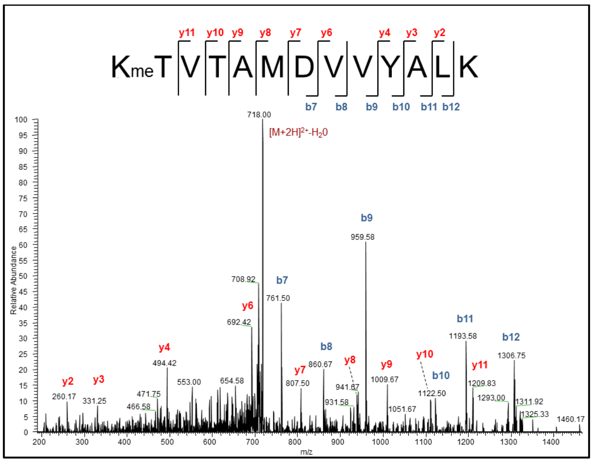

me2 at K79

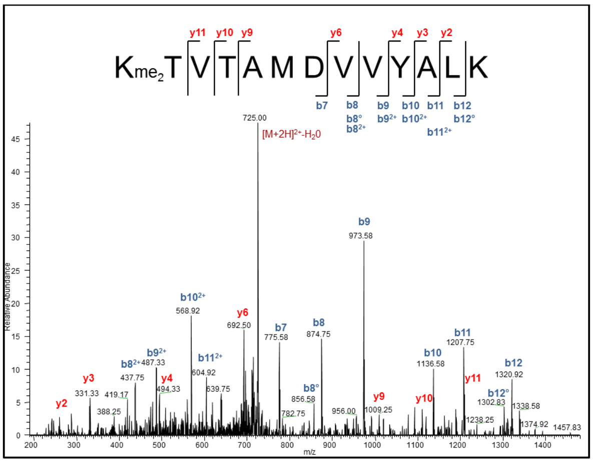

H2B

Ac at K2, K6, K10

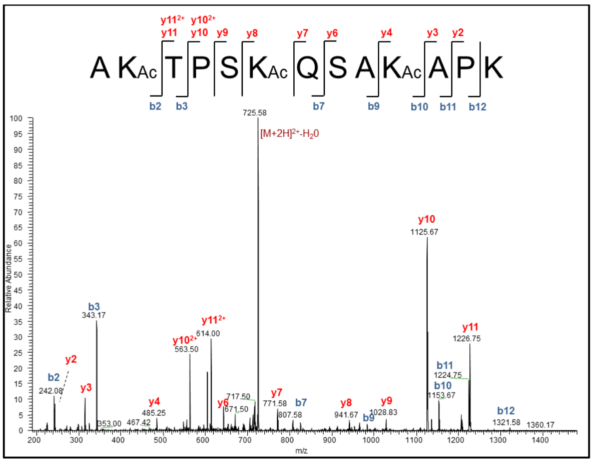

Ac at K34

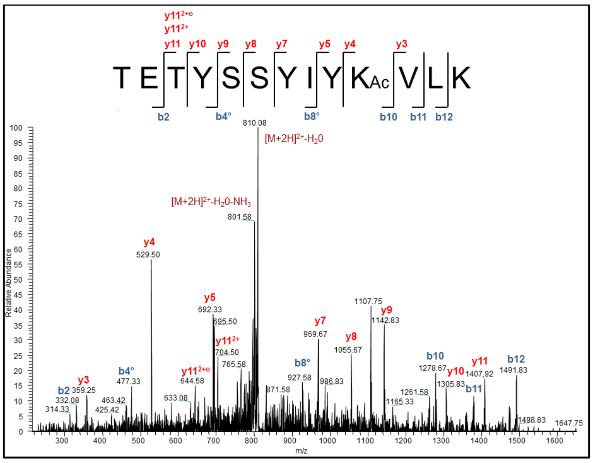

Ac at K107

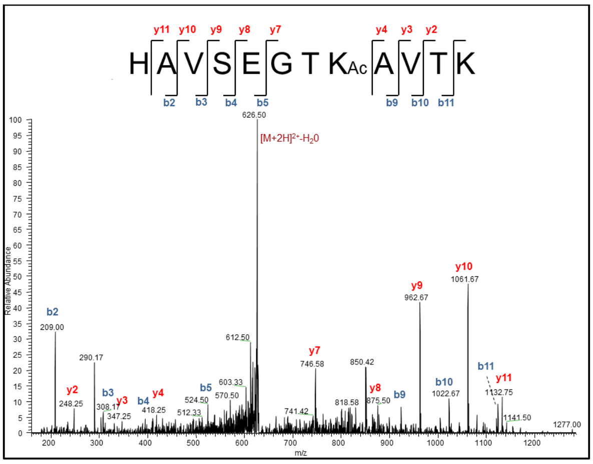

Ub at K111

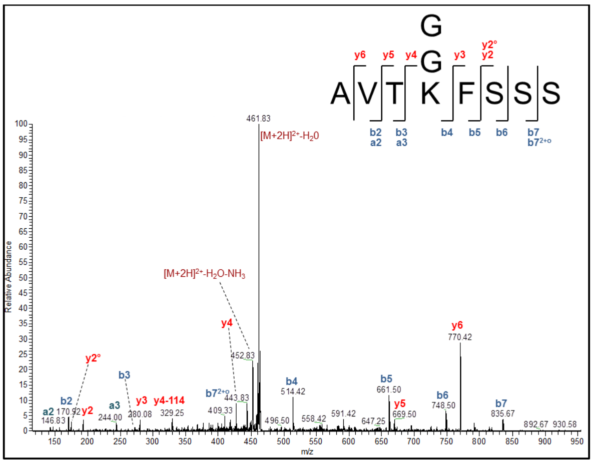

H3

Ac at K9, K14

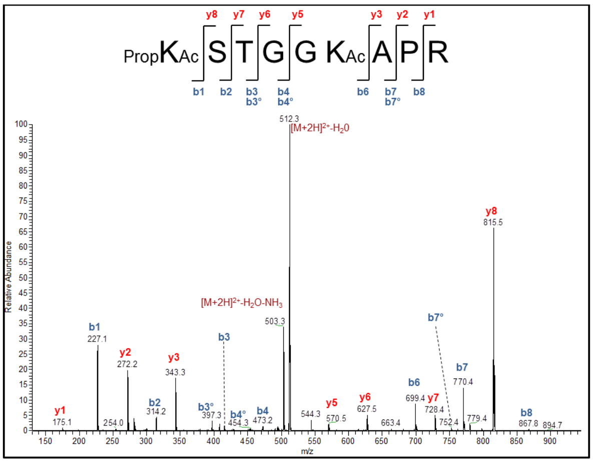

Ac at K9, K14 me3 at K4

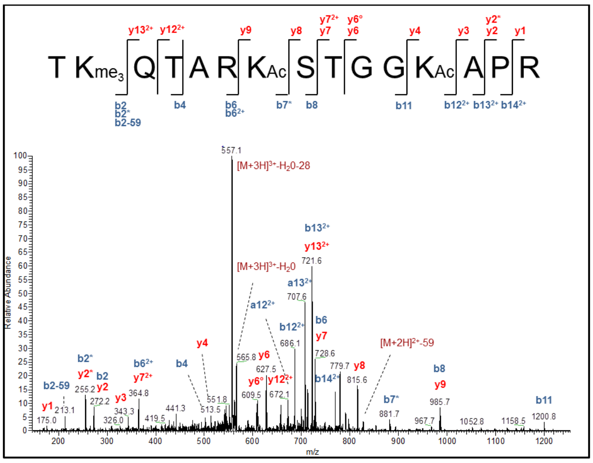

Ac at K9, K14 me2 at K4

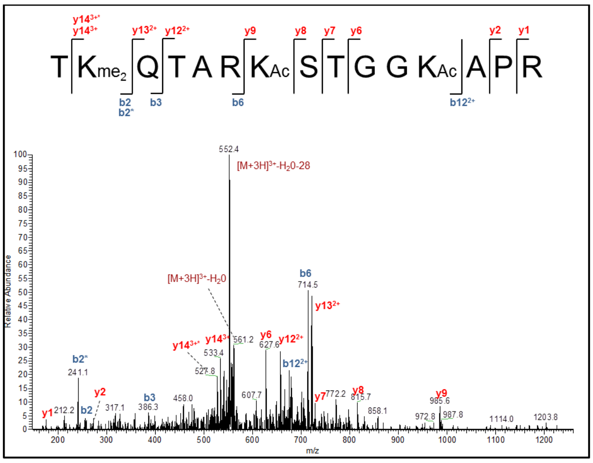

me3 at K27

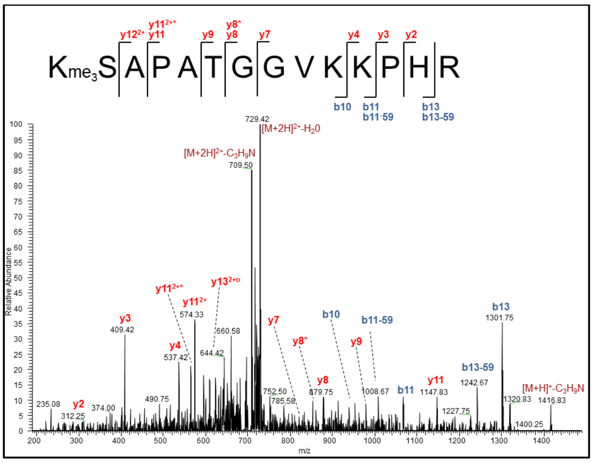

H4

Ac at K59

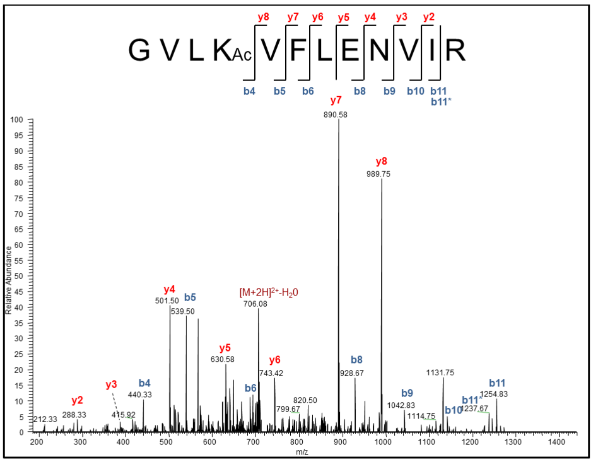

me at K79

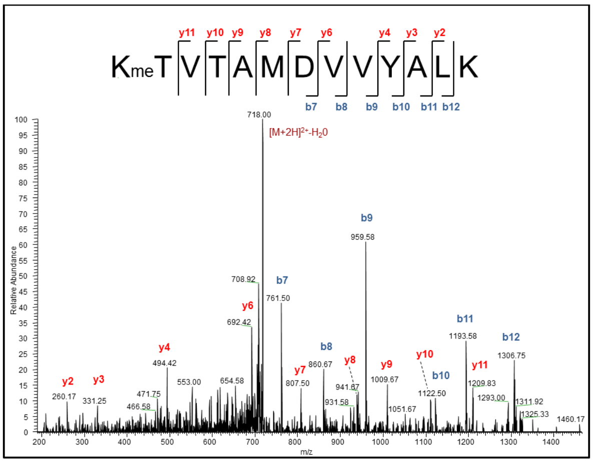

me2 at K79

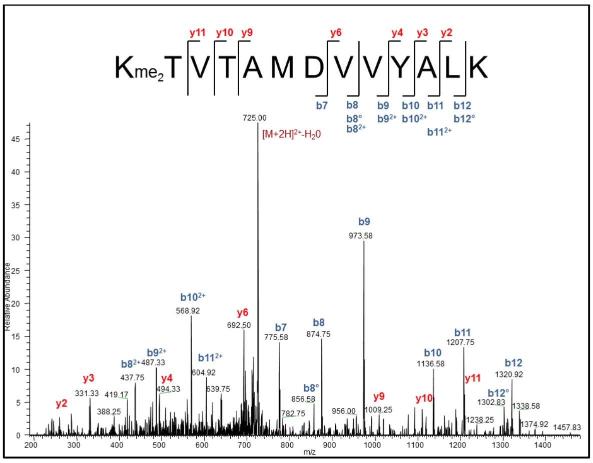

Supplement: Additional file 2: Figure S2. — Identification of histone modification sites. MS/MS spectrum of novel PTMs detected in histones H3, H4, H2A and H2B, as well as histone PTMs that were mapped. [file 13059_2015_671_MOESM2_ESM.pdf]
