# Supplementary material for: An integrative analysis of post-translational histone modifications in the marine diatom Phaeodactylum tricornutum
Source: Genome Biol. 2015 May 20;16(1):102. doi: 10.1186/s13059-015-0671-8 (PMC4504042; doi:10.1186/s13059-015-0671-8)
Supplement: Additional file 16: Table S5. — The sites of post-tanslational modifications (PTMs) on histones include amino acid (residue) and peptide sequences which are acetylated (Acetyl), methylated (Methyl, Dimethyl and Trimethyl) or ubiquitinated. [file 13059_2015_671_MOESM16_ESM.pdf]

| H4 | residue | modification (PTM) | Trypsine<br>Peptide sequence and modified amino acid | mgf file | Mascot Score |
|---|---|---|---|---|---|
| | K5, K8, K12 and K16 | Acetyl | GKGGKGLGKGGAKR + Acetyl (K:2.5.9.13) | F8001BL | 76,23 |
| | K20 | Acetyl | KVLRDNIQGITKPAIR + Acetyl (K:1) | F8457BL | 40,17 |
| | K31 | Acetyl | DNIQGITKPAIR + Acetyl (K:8) | F8001BL | 35,73 |
| | K59 | Acetyl | GVLKVFLENVIR + Acetyl (K:4) | F7771BL | 52,13 |
| | K59 | Methyl | GVLKVFLENVIR + Methyl (K:4) | F7771BL | 60,6 |
| | K79 | Methyl | KTVTAMDVVYALK + Methyl (K:1) + Oxidation (M:6) | F7638BL | 66,19 |
| | K79 | Dimethyl | KTVTAMDVVYALK + Dimethyl (K:1) | F8001BL | 63,16 |
| | K79 | Trimethyl | KTVTAMDVVYALK + Oxidation (M:6) + Trimethyl (K:1) | F7638BL | 62,58 |
| | | | Chymotrypsine | | |
| | N-terminus, K5 and K8 | Acetyl | SGRGKGGKGL + Acetyl (K:5.8) + Acetyl (N-term) | F7672BL | 38,55 |
| | | | ArgC | | |
| | K5, K8, K12 and K16 | Acetyl | GKGGKGLGKGGAKR + Acetyl (K:2.5.9.13) | F8374BL | 89,07 |
| | K20 | Acetyl | KVLRDNIQGITKPAIR + Acetyl (K:1) | F8374BL | 52,73 |
| | K79 | Methyl | KTVTAMDVVYALKR + Methyl (K:1) | F8374BL | 56,23 |
| | K79 | Dimethyl | KTVTAMDVVYALKR + Dimethyl (K:1) | F8374BL | 65,86 |

| H2B | residue | modification (PTM) | Trypsine<br>peptide sequence and modified amino acid | mgf file | Mascot Score |
|---|---|---|---|---|---|
| | K2 | Acetyl | AKTPSKQSAKAPK + Acetyl (K:2.6.10) | F8001BL | 62,35 |
| | K6 | Acetyl | AKTPSKQSAKAPK + Acetyl (K:2.6.10) | F8001BL | 63,35 |
| | K10 | Acetyl | AKTPSKQSAKAPK + Acetyl (K:2.6.10) | F8001BL | 64,35 |
| | K10, K13 and K14 | Acetyl | QSAKAPKKAATGTK + Acetyl (K:4.7.8) | F7639BL | 67,4 |
| | K34 | Acetyl | TETYSSYIYKVLK + Acetyl (K:10) | F8001BL | 45,63 |
| | K107 | Acetyl | HAVSEGTKAVTK + Acetyl (K:8) | F8001BL | 48,97 |
| | K111 | Ubiquitin | AVTKFSSS + GlyGly (K:4) | F7427FD | 31,33 |
| | | | Propionylation + trypsine | | |
| | K2 | Acetyl | AKTPSKQSAKAPK + Acetyl (K:2) + Propionyl (K:6.10) + Propionyl (N-term) | F7677BL | 37,16 |
| | K6 | Acetyl | AKTPSKQSAKAPKK + Acetyl (K:6) + Propionyl (K:2.10.14) + Propionyl (N-term) | F7677BL | 31,82 |
| | K10 | Acetyl | QSAKAPKKAATGTK + Acetyl (K:4) + Propionyl (K:7.8) | F7677BL | 32,04 |
| | K13 and K14 | Acetyl | QSAKAPKKAATGTK + Acetyl (K:7.8) + Propionyl (K:4) | F7677BL | 29,3 |
| | K48 | Acetyl | KGMSIMNSFINDIFER + Acetyl (N-term) + Oxidation (M:6) + Propionyl (K:1) | F7677BL | 41,47 |

| H2A.Z | residue | modification (PTM) | Trypsine<br>peptide sequence and modified amino acid | mgf file | Mascot Score |
|---|---|---|---|---|---|
| | N-terminus, K3, K6, K9, K12 and K15 | Acetyl | SGKGGKGGKGGKGGKAPTSK + Acetyl (K:3.6.9.12.15) + Acetyl (N-term) | F8001BL | 55,25 |
| | | | ArgC | | |
| | N-terminus, K3, K6, K9, K12 and K15 | Acetyl | SGKGGKGGKGGKGGKAPTSKKAPQSR + Acetyl (K:3.6.9.12.15) + Acetyl (N-term) | F8025BL | 44,08 |

| H3 | residue | modification (MPT) | Trypsine<br>Peptide sequence and modified amino acid | mgf file | Mascot Score |
|---|---|---|---|---|---|
| | K18 and K23 | Acetyl | KQLATKAAR + Acetyl (K:1.6) | F7639BL | 65,11 |
| | K27 | Acetyl | KSAPATGGVK + Acetyl (K:1) | F7397FD | 52,35 |
| | K27 and K36 | Methyl | KSAPATGGVKKPHR + Methyl (K:1.10) | F7771BL | **27,16** |
| | K27 | Dimethyl | KSAPATGGVKKPHR + Dimethyl (K:1) + Methyl (K:10) | F8001BL | 47,19 |
| | K27 | Trimethyl | KSAPATGGVKKPHR + Methyl (K:10) + Trimethyl (K:1) | F7396FD | **24,59** |
| | K36 | Acetyl | SAPATGGVKKPHR + Acetyl (K:9) | F8001BL | 42,9 |
| | K36 | Methyl | SAPATGGVKKPHR + Methyl (K:9) | F7639BL | 52,09 |
| | K36 | Dimethyl | SAPATGGVKKPHR + Dimethyl (K:9) | F8001BL | 58,82 |
| | K36 | Trimethyl | SAPATGGVKKPHR + Trimethyl (K:9) | F7396FD | **28,86** |
| | K56 | Acetyl | YQKSTDLLIR + Acetyl (K:3) | F7981BL | **41,12** |
| | K79 | Acetyl | EIAQDFKSDLR + Acetyl (K:7) | E7981BL | 67,24 |
| | K79 | Methyl | EIAQDFKSDLR + Methyl (K:7) | F8001BL | **33,46** |
| | K79 | Dimethyl | EIAQDFKSDLR + Dimethyl (K:7) | F8001BL | 35,73 |
| | K122 | Acetyl | VTIMPKDIQLAR + Acetyl (K:6) + Oxidation (M:4) | F7981BL | 52,39 |

| residue | modification (MPT) | ArgC<br>Peptide sequence and modified amino acid | mgf file | Mascot Score |
|---|---|---|---|---|
| K4 | Dimethyl | TKQTARKSTGGKAPR + Acetyl (K:7.12) + Dimethyl (K:2) | F8025BL | **24,63** |
| K4 | Trimethyl | TKQTARKSTGGKAPR + Acetyl (K:7.12) + Trimethyl (K:2) | F7982BL | **23,6** |
| K9, K14, K18 and K23 | Acetyl | KSTGGKAPRKQLATKAAR + Acetyl (K:1.6.10.15) | F8375BL | 32,03 |
| K18 and K23 | Acetyl | KQLATKAAR + Acetyl (K:1.6) | F8375BL | 51,87 |
| K27 | Acetyl | KSAPATGGVKKPHR + Acetyl (K:1) | F8375BL | 34,19 |
| K27 | Trimethyl | KSAPATGGVKKPHR + Trimethyl (K:1) | F8375BL | 43,21 |
| K36 | Methyl | KSAPATGGVKKPHR + Methyl (K:10) | F8375BL | 47,49 |
| K36 | Dimethyl | KSAPATGGVKKPHR + Dimethyl (K:10) | F8375BL | 57,99 |
| K79 | Methyl | LVREIAQDFKSDLR + Methyl (K:10) | F8375BL | 45,32 |
| K79 | Dimethyl | LVREIAQDFKSDLR + Dimethyl (K:10) | F8375BL | **27,22** |
| K122 | Acetyl | VTIMPKDIQLAR + Acetyl (K:6) + Oxidation (M:4) | F7982BL | **25,17** |

| residue | modification (MPT) | Propionylation + trypsine<br>Peptide sequence and modified amino acid | mgf file | Mascot Score |
|---|---|---|---|---|
| K9 | Acetyl | KSTGGKAPR + Acetyl (K:1) + Propionyl (K:6) | F8535BL | 45,25 |
| K9 | Methyl | KSTGGKAPR + Acetyl (K:6) + Acetyl (N-term) + Methyl (K:1) | F8535BL | 44,24 |
| K18 and K23 | Acetyl | KQLATKAAR + Acetyl (K:1.6) | F7677BL | 38,59 |
| K27 | Acetyl | KSAPATGGVKKPHR + Acetyl (K:1) + Propionyl (K:10.11) | F7677BL | 42,14 |
| K27 | Dimethyl | KSAPATGGVKKPHR + Dimethyl (K:1) + Propionyl (K:10.11) | F7677BL | 56,89 |
| K27 | Trimethyl | KSAPATGGVKKPHR + Propionyl (K:10.11) + Trimethyl (K:1) | F7677BL | 75,62 |
| K36 | Methyl | SAPATGGVKKPHR + Methyl (K:9) + Propionyl (K:10) | F7677BL | 58,02 |
| K36 | Dimethyl | SAPATGGVKKPHR + Dimethyl (K:9) + Propionyl (K:10) | F7677BL | 43,49 |
| K79 | Dimethyl | EIAQDFKSDLR + Dimethyl (K:7) | F7677BL | **27,23** |

| H2A | residue | modification (PTM) | peptide sequence and modified amino acid | mgf file | Mascot Score |
|---|---|---|---|---|---|
| | | | Trypsine | | |
| | N-terminus, K3, K5 and K7 | Acetyl | SGKGKGKGGR + Acetyl (K:3.5.7) + Acetyl (N-term) | F7771BL | 46,69 |
| | | | ArgC | | |
| | N-terminus, K3, K5 and K7 | Acetyl | SGKGKGKGGR + Acetyl (K:3.5.7) + Acetyl (N-term) | F8374BL | 39,82 |
